# Dissecting metabolic dysfunction- and alcohol-associated liver disease (MetALD) using proteomic and metabolomic profiles

**DOI:** 10.1016/j.jhep.2025.05.026

**Published:** 2025-06-04

**Authors:** Jun Liu, Sihao Xiao, Sile Hu, Rui Huang, Lingyan Chen, Jeremy W. Tomlinson, Jeremy F. Cobbold, Joanna M.M. Howson, Cornelia M. van Duijn

**Affiliations:** 1 Nuffield Department of Population Health, University of Oxford, Oxford, UK; 2 Genetics Centre-of-Excellence, Novo Nordisk Research Centre Oxford, Oxford, UK; 3 Oxford Centre for Diabetes, Endocrinology and Metabolism, University of Oxford, Churchill Hospital, Oxford, UK; 4 NIHR Oxford Biomedical Research Centre, Oxford University Hospitals NHS Foundation Trust and the University of Oxford, Oxford, UK; 5 Translational Gastroenterology and Liver Unit, University of Oxford, Oxford, UK

**Keywords:** Steatotic liver disease, MetALD, MASLD, ALD, metabolomics, proteomics, classification

## Abstract

**Background & Aims:**

Metabolic dysfunction- and alcohol-associated liver disease (MetALD) is a poorly understood condition that bridges cardiometabolic and alcohol-related pathological characteristics. We aimed to differentiate patients with MetALD whose molecular signatures more closely resemble either alcohol-related liver disease (ALD) or metabolic dysfunction-associated steatotic liver disease (MASLD), and to assess their relative risks of complications and mortality.

**Methods:**

We analysed data from 443,453 European participants in the UK Biobank, including 34,147 with MetALD, 11,220 with ALD, and 124,034 with MASLD. Elastic net regression was used to classify ALD and MASLD based on 249 plasma metabolites and/or 2,941 plasma proteins, with multiple sensitivity analyses. We then applied the resulting concise model to patients with MetALD to identify an alcohol-predominant group (classified as ALD) and a cardiometabolic-predominant group (classified as MASLD). Finally, we evaluated their 15-year risk of major outcomes (heart failure, myocardial infarction, stroke, cirrhosis, hepatocellular carcinoma, and mortality) using Cox regression.

**Results:**

The metabolome alone discriminated ALD from MASLD with an AUC of 0.86, while the proteome alone achieved an AUC of 0.96. Adding age, sex, BMI, liver enzymes, or metabolome information did not enhance the AUC of the proteome model. A 10-protein model differentiated ALD from MASLD with an AUC of 0.93. This model identified that patients with alcohol-predominant MetALD had significantly higher risks of mortality, and cirrhosis, along with elevated fibrosis scores and higher fibrosis stages, compared to patients with cardiometabolic-predominant MetALD.

**Conclusions:**

This study highlights the value of proteomic subtyping in MetALD, enabling more personalized treatment strategies and improved prognostic assessment.

## Graphical abstract

**Graphical abstract:**
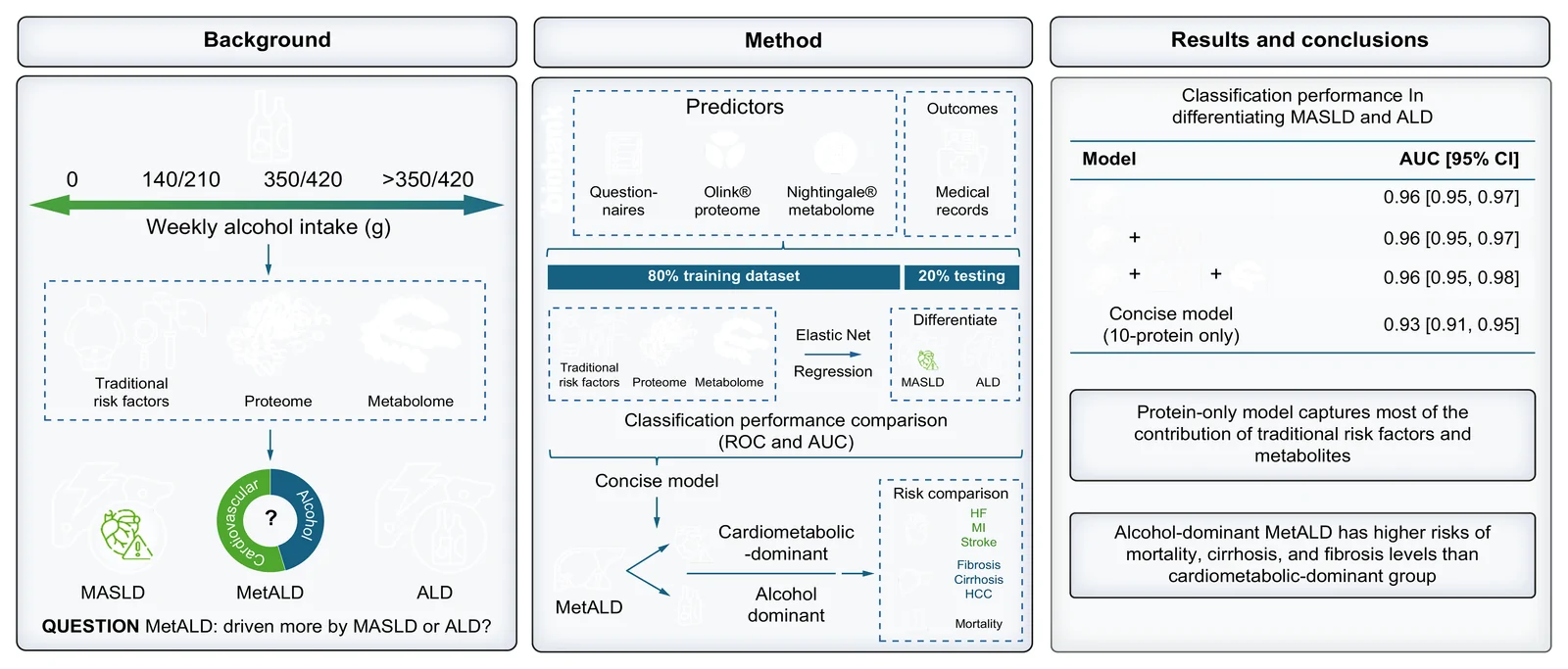

## Introduction

Metabolic dysfunction- and alcohol-associated liver disease (MetALD) is defined by the presence of metabolic dysfunction-associated steatotic liver disease (MASLD) coupled with increased alcohol intake (between 140–350 g/week for females and 210–420 g/week for males). It represents a continuum that can be dominated by either MASLD or by alcohol-related liver disease (ALD).^1^ Within this continuum, certain individuals exhibit prominent cardiometabolic features and an elevated risk of cardiovascular disease, while others show a predominance of alcohol-related liver pathology and a higher risk of adverse hepatic outcomes, such as cirrhosis and hepatocellular carcinoma.^1^

As a newly defined disease category in 2023, research on MetALD is limited. Experts have emphasized the need to distinguish ALD-dominant from MASLD-dominant MetALD.^2^ However, clear criteria for making this distinction are lacking, and the extent to which alcohol intake influences the pathology and prognosis of MetALD compared to MASLD remains uncertain.^1^ Moreover, there are no adequately validated objective tools to assess the contributions of MASLD and ALD, resulting in reliance on self-reported alcohol intake.^1^ Given the unreliability of patients’ recall of alcohol consumption^3^ and the tendency for individuals to modify their drinking behaviour due to health problems or concerns,^4^ misclassification of ALD and MetALD as MASLD is common, and risks for major liver outcomes, such as cirrhosis, may go unrecognized.^5^

The liver plays a crucial role in metabolizing proteins, metabolites, and alcohol. Therefore, protein and metabolite profiles can provide valuable insights into the effects of alcohol in patients with MASLD, ALD and MetALD. Differentiating the proteome and metabolome profiles of patients with MASLD and ALD may uncover molecular patterns that are associated with either metabolic- or alcohol-related pathology, helping to identify patients whose condition is primarily driven by alcohol or by metabolic dysfunction.

In this study, we used molecular profiling, including plasma proteome and metabolome data, to classify patients with MetALD based on whether ALD or MASLD was the dominant disease driver. We then assessed the relative risks of major complications and mortality within these subgroups.

## Patients and methods

### Study population

We conducted our study using data from the UK Biobank, a large prospective cohort comprising over 500,000 participants aged 37 to 73 years at the time of recruitment (2006–2010). Following a standardized protocol, data collection included self-reported questionnaires, physical and anthropometric measurements, clinical biomarkers, metabolite and protein assays, and linked electronic health records. All participants provided electronically signed informed consent. The UK Biobank study received ethical approval from the North West Multi-centre Research Ethics Committee, the Patient Information Advisory Group, and the Community Health Index Advisory Group. The present analysis is part of UK Biobank projects 53639 and 65851. Additional details about the UK Biobank are available online.^6^

**Fig. 1:**
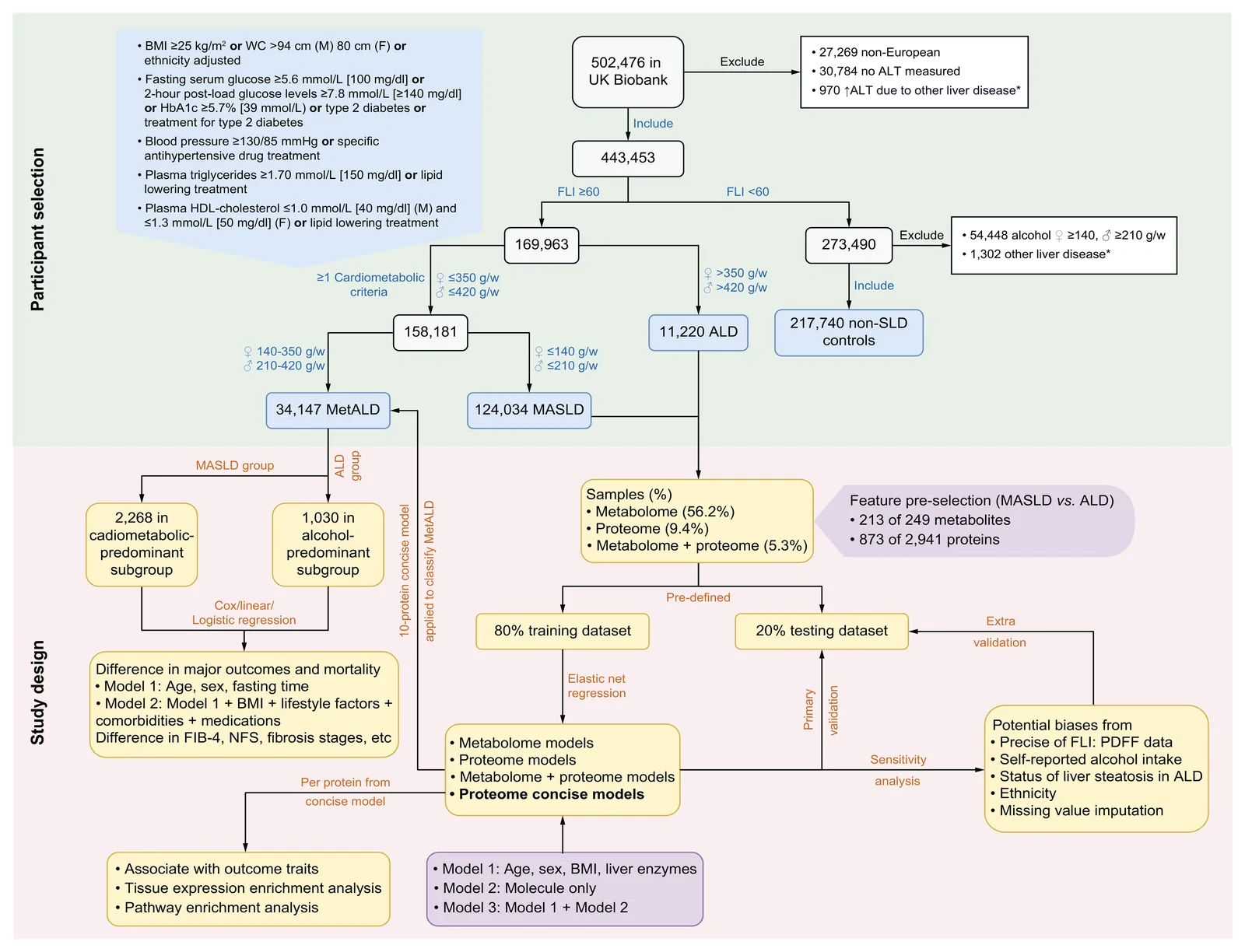
Flowchart of participant selection and study design. * Other causes of chronic liver disease include viral hepatitis, autoimmune liver disease, genetic liver disorders, biliary cirrhosis, and sarcoidosis. ♀F Female. ♂M Male. ALD, alcohol-related liver disease; ALT, alanine aminotransferase; FLI, fatty liver index; MASLD, metabolic dysfunction-associated steatotic liver disease; MetALD, metabolic dysfunction- and alcohol-associated liver disease; (MRI-)PDFF, MRI-estimated proton density fat fraction; SLD, steatotic liver disease. (This figure appears in color on the web.)

In the present study, we limited our analysis to participants of European ancestry who had available alanine aminotransferase (ALT) measurements. We excluded individuals with elevated liver enzymes (ALT >33 IU/L for males or ALT >25 IU/L for females) due to other liver disease causes at baseline, including viral hepatitis, autoimmune liver disease, genetic liver disorders, biliary cirrhosis, and sarcoidosis.^1,7^ This resulted in a cohort of 443,453 participants. We also limited our analysis to participants with metabolome (n = 261,638) and proteome (n = 41,767) data available for relevant analyses. The flowchart for participant selection is presented in Fig. 1.

### Definitions of MASLD, MetALD and ALD and related traits

We used fatty liver index (FLI) as an indicator of hepatic steatosis, following the standard approach used in large-scale epidemiological studies.^8–10^ We defined steatotic liver disease (SLD) as having an FLI ≥60 at baseline (referred to as FLI-based diagnosis).^8^ Recognizing limitations in the accuracy of FLI, we performed a sensitivity analysis by defining SLD through MRI-estimated proton density fat fraction (MRI-PDFF) ≥5, which was measured during the second instance of the UK Biobank (referred to as PDFF-based diagnosis).^11^ MASLD was defined in patients with SLD who met at least one of the five cardiometabolic criteria at baseline, as outlined in the latest guidelines for MASLD (Fig. 1).^11^ Alcohol consumption was obtained at baseline using a questionnaire that detailed average weekly consumption of various alcoholic beverages. According to the guidelines,^11^ patients were categorised as having MASLD (≤140 g/week for females, ≤210 g/week for males), MetALD (140–350 g/week for females, 210–420 g/ week for males), or ALD (>350 g/week for females, >420 g/ week for males). The non-SLD control group consisted of participants who did not have SLD, had no other underlying chronic liver disease at baseline, and consumed less than 140 g/week for females and 210 g/week for males (Fig. 1). Other underlying chronic liver diseases were defined based on self-report at baseline (Table S1). The fibrosis stage was determined based on the fibrosis-4 index (FIB-4) and NAFLD fibrosis score (NFS). Patients were classified as having fibrosis stage 2 (n = 17,260) if their FIB-4 was greater than 2.67 or NFS was greater than 0.675. Fibrosis stage 0 (n = 222,227) was defined by FIB-4 less than 1.3 or NFS less than -1.455. The remaining patients, with values between stage 0 and stage 2, were assigned to fibrosis stage 1 (n = 228,846).^12^ The definitions of other relevant covariates, including BMI, blood biochemical biomarkers, fasting time, type 2 diabetes, hypertension, medication information, smoking status, smoking pack-years, education, and physical activity, are provided in Table S2. For missing values in the variables used to define FLI and the five cardiometabolic criteria for MASLD,^1^ we used chained random forests through the R package *missRanger* for missing value imputation (Supplementary Methods).

### Definitions of major outcomes

We studied the major outcomes of SLD leading to death, which included 1) cardiovascular outcomes (*i.e*. heart failure, myocardial infarction, and stroke), and 2) major hepatic outcomes (*i.e.* cirrhosis and hepatocellular carcinoma), and all-cause mortality. We defined incident events and onset dates based on self-reported illness, hospital inpatient records, or death register records up until November 2022. The earliest recorded date of a disease was used as the date of disease diagnosis. Prevalent cases were defined as participants who had either a disease diagnosis date before or on the first assessment date or who self-reported the condition on the first assessment date. The censor date was defined as the earliest of the following: the first recorded date of the event, the death date, or the end of the digital recording period. The detailed ICD codes and self-reported codes of the cardiovascular and hepatic outcomes are shown in Table S1. All-cause mortality was based on death registration records.

### Metabolome and proteome measurements

Plasma metabolites in the UK Biobank were quantified using a targeted high-throughput ^1^H-nuclear magnetic resonance metabolomics platform provided by Nightingale Health Ltd (biomarker quantification version 2020).^13^ This platform simultaneously measures 249 metabolites, including clinical lipids, detailed lipoprotein subclass profiles, fatty acid composition, and a variety of small molecules, such as amino acids, ketone bodies, and glycolysis-related metabolites. The method follows a standardized protocol for sample quality control, preparation, data storage, and automated spectral analysis.^13,14^ A map of all metabolites and their full names is shown in Table S3.

High-throughput proteomics measurements were performed by Olink^®^.^15^ We included the 46,673 randomly selected participants for the proteome analysis. Details of the Olink proteomics assay, data processing and quality control have been described previously.^15^ We included 2,941 protein values of their Normalized Protein eXpression (NPX), which are measured in Olink’s arbitrary log2 scale.^15^ A map of all protein variables and their full names is provided in Table S4.

### Statistical analysis

All analyses were performed using R statistical software (version 4.0.3), and two-tailed tests were applied.

We used logistic regression, adjusting for age, sex, and fasting time, to associate clinical and molecular features with binary outcomes. Bonferroni correction was applied for multiple testing adjustments using the matrix spectral decomposition method to control for the correlation among testing features.^16^ This included 25 independent tests for 33 commonly known SLD-related risk factors (*p* <2.0×10^-3^, 0.05/25), 38 for 249 metabolites (*p* <1.3×10^-3^, 0.05/38), and 1,428 for 2,941 proteins (*p* <3.5×10^-5^, 0.05/1,428). Cox proportional hazards model (R package *survival*) was used to estimate the relationship between baseline exposures and the risk of incident events during follow-up. Participants with the prevalent outcome were excluded from the survival analysis. We also performed a sensitivity analysis that removed all prevalent major outcomes at baseline to mitigate potential biases from log-term outcomes. Competing-risk regression based on Fine and Gray’s method (R package *cmprsk*) was used as a sensitivity analysis considering the competing risk of mortality. The proportional hazards assumption was verified for all analyses.

To differentiate ALD and MASLD, participants were randomly divided into a training dataset (80%) and a testing dataset (20%). All protein and metabolite features have less than 20% missing data, which were imputed with the mean value for each feature. The protein and metabolite values were then rank-based inverse normal transformed before applying the classification models. In the training dataset, we applied elastic net regression for models with multiple features, and logistic regression for models with a single feature. Model training employed repeated stratified 5-fold cross-validation (three iterations), synthetic minority over-sampling technique for resampling, and the receiver-operating characteristic curve metric for tuning parameter selection, using the *caret* package in R. Three classification models were developed: Model 1 included age, sex, BMI, and liver enzymes (ALT, aspartate aminotransferase [AST], gamma-glutamyltransferase [GGT], and AST/ALT ratio); Model 2 included only molecules that were significantly different between ALD and MASLD; Model 3 combined Model 1 and Model 2. The ROC and predictive performance (AUC, specificity, and sensitivity) were assessed using participants in the predefined independent testing dataset. To develop a concise model with strong discrimination capability yet a limited number of proteins, we employed a forward stepwise feature selection method. We iteratively added the most significant protein based on the individual classification performance at each step to improve the model’s performance until it reached a plateau. We then tested additional classification models among all possible permutations of the top proteins to determine whether any combinations with fewer proteins could yield a higher AUC than the combination of all the top proteins. Various sensitivity analyses were performed for the classification models to address potential biases arising from the method used to define liver steatosis, self-reported alcohol intake, the status of liver steatosis in ALD, ethnicity, and the imputation of missing values (Supplementary Methods). The probability threshold for specificity was set at 0.95, allowing a false positive rate as low as 5% in the ALD-MASLD classification model. This threshold was used to distinguish MetALD with alcohol-predominant characteristics (classified as ALD) from MetALD with cardiometabolic-predominant characteristics (classified as MASLD). We followed this with survival analysis and Cox regression to evaluate patients’ 15-year risk of major prognostic complications and mortality during follow-up. This included a basic model adjusted for age, sex, and fasting time, and an advanced model additionally adjusted for BMI, lifestyle factors (smoking status, pack-years, education, and physical activity), baseline cardiometabolic comorbidities (type 2 diabetes, and hypertension) and medications (anti-diabetes, anti-hypertensives and lipid-lowering medications).

To explore the potential mechanistic roles of the 10 identified proteins in relation to diseases, we used the FUMA GEN-E2FUNC pipeline.^17^ We looked at the gene expression levels using log2 transformed average expression values and conducted enrichment analysis based on pre-calculated differentially expressed gene sets across 30 different general tissue types. Genes with log2 transformed expression values exceeding 2.84 – approximately 6.5-fold higher than the median expression level across all genes – were classified as highly expressed. Additionally, we examined whether these 10 proteins were enriched in any pathways using all gene types as the background.^17^

## Results

### Clinical profile of MetALD comparing with ALD and MASLD

We identified 34,147 (7.7%) participants with MetALD, 124,034 (28.0%) with MASLD, and 11,220 (2.5%) with ALD (Fig. 1). The non-SLD control group consisted of 217,740 participants (49.1%). Compared to the non-SLD group, patients with SLD showed significantly higher cardiometabolic risk factors, less favourable lifestyle habits, and elevated liver enzyme and liver steatosis levels (Table 1, Fig. S1, *p* <2.0×10^-3^). Patients with MetALD exhibited characteristics intermediate between those of patients with MASLD and ALD. Compared to patients with MASLD, those with ALD had higher HDL-cholesterol, liver enzymes, blood pressure, and a higher rate of smoking and liver steatosis. However, they were younger, had lower education, BMI, waist circumference, and a reduced risk of type 2 diabetes. When replicating the analysis using the PDFF-based diagnosis, we observed a similar pattern, though the significance levels were less pronounced (Fig. S1; Supplementary Results).

**Fig. 2:**
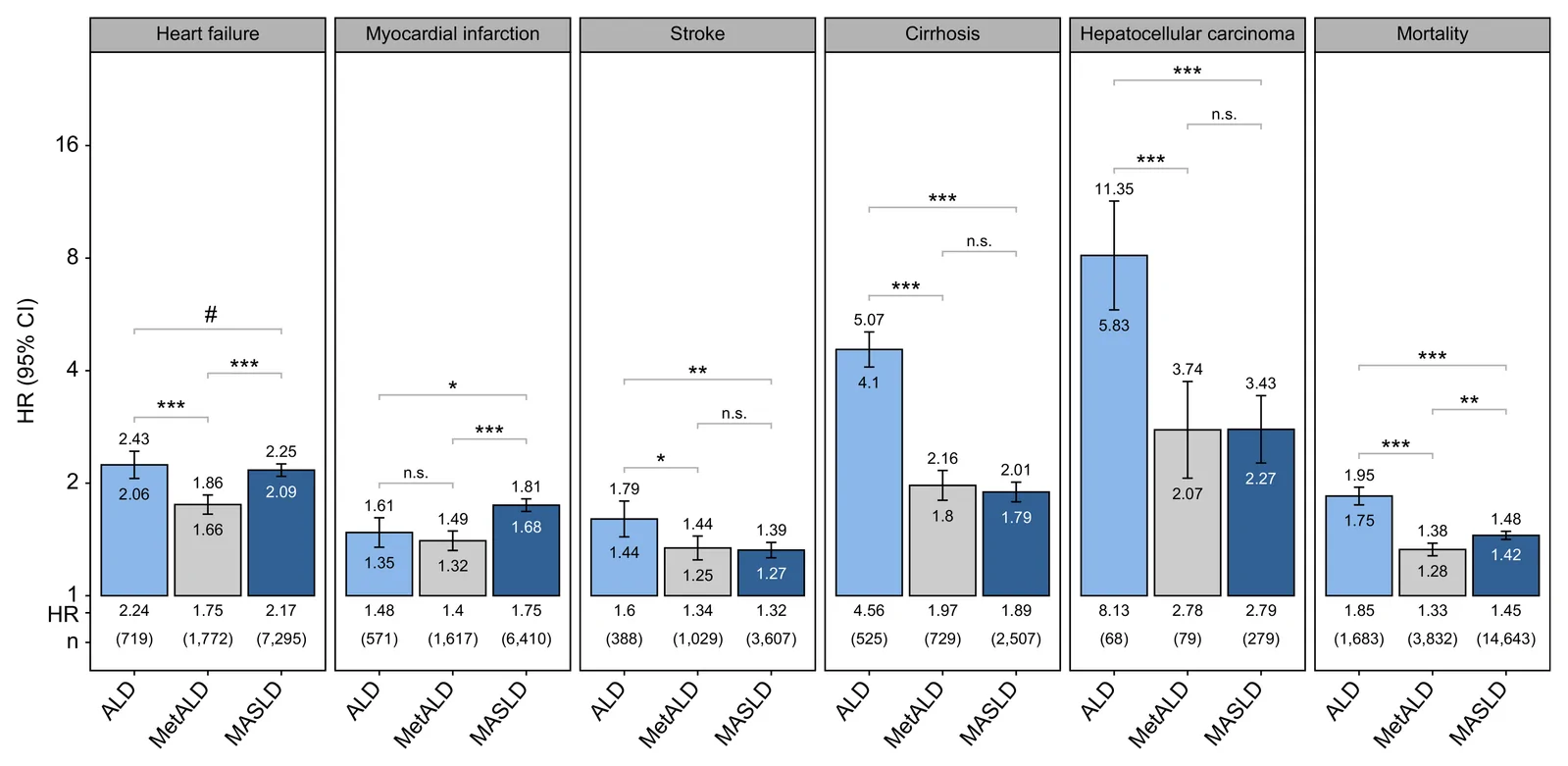
HR of major outcomes in patients with ALD, MetALD and MASLD, compared to non-SLD controls. Cox regression was performed with adjustments for age, sex, and fasting time. The HR values are positioned beneath the bar plots, accompanied by 95% CI adjacent to the error bars. Number of incident outcome events in each case group is denoted by “n” in the brackets. ***p <0.0001, **p <0.001, *p <0.0028, #*p*<0.05, n.s. *p ≥*0.05. ALD, alcohol-related liver disease; HR, hazard ratio; MASLD, metabolic dysfunction-associated steatotic liver disease; MetALD, metabolic dysfunction- and alcohol-associated liver disease; SLD, steatotic liver disease.

We investigated the risk of major outcomes, *i.e.* three cardiovascular outcomes, two hepatic outcomes and mortality, in patients with different SLD subtypes during a follow-up period of up to 15 years. Fig. 2 and S2 show that patients with SLD had a significantly higher risk for cardiovascular events, hepatic events, and mortality compared to those without SLD (*p* <2.8 ×10^-3^). Compared to patients with MetALD, those with ALD had a higher risk of mortality, cirrhosis, hepatocellular carcinoma, stroke, and heart failure; those with MASLD had a higher risk of mortality, heart failure and myocardial infarction. A similar pattern was observed with the PDFF-based approach for stroke, cirrhosis, and mortality, although the results were not statistically significant due to small sample sizes (Supplementary Results; Fig. S3). No significant difference was detected between MetALD and ALD for myocardial infarction, or between MetALD and MASLD for cirrhosis, hepatocellular carcinoma, and stroke. These significant results persisted after accounting for the competing risk of mortality (Fig. S4) or excluding participants with any prevalent major outcomes (Fig. S5).

### Molecular classification of ALD and MASLD

Fig. 1 shows the major study design. We developed classification models in the training dataset using traditional risk factors and pre-selected molecules to differentiate between MASLD and ALD. We then tested these models on a separate testing dataset and performed sensitivity analyses for bias. The concise protein model was used to categorise patients with MetALD into alcohol-driven and cardiometabolic-driven subgroups. Finally, we assessed clinical progression of these subgroups and analysed the role of the 10 selected proteins individually.

Compared to those with MASLD, patients with MetALD had significantly higher levels of 136 metabolites (54.6%), including increased levels of 45 HDL subtypes, and lower levels of 77 metabolites (30.9%), which comprised seven amino acid variables, 36 VLDL subtypes, and various lipids (*p* <1.3×10^-3^; Table S5). When comparing protein abundance between ALD and MASLD, we identified 319 proteins (10.8%) that were significantly increased in patients with ALD, and 554 proteins (18.8%) that were significantly increased in patients with MASLD (*p* <3.5×10^-5^; Table S6). These findings covered all the significant associations from the replication analysis under the PDFF-based diagnosis (Tables S7–8). Strong correlations were also found in effect estimates of associations between the PDFF-based and FLI-based diagnoses for metabolites (*r* = 0.98) and proteins (*r* = 0.72; Supplementary Results; Fig. S6).

**Fig. 3:**
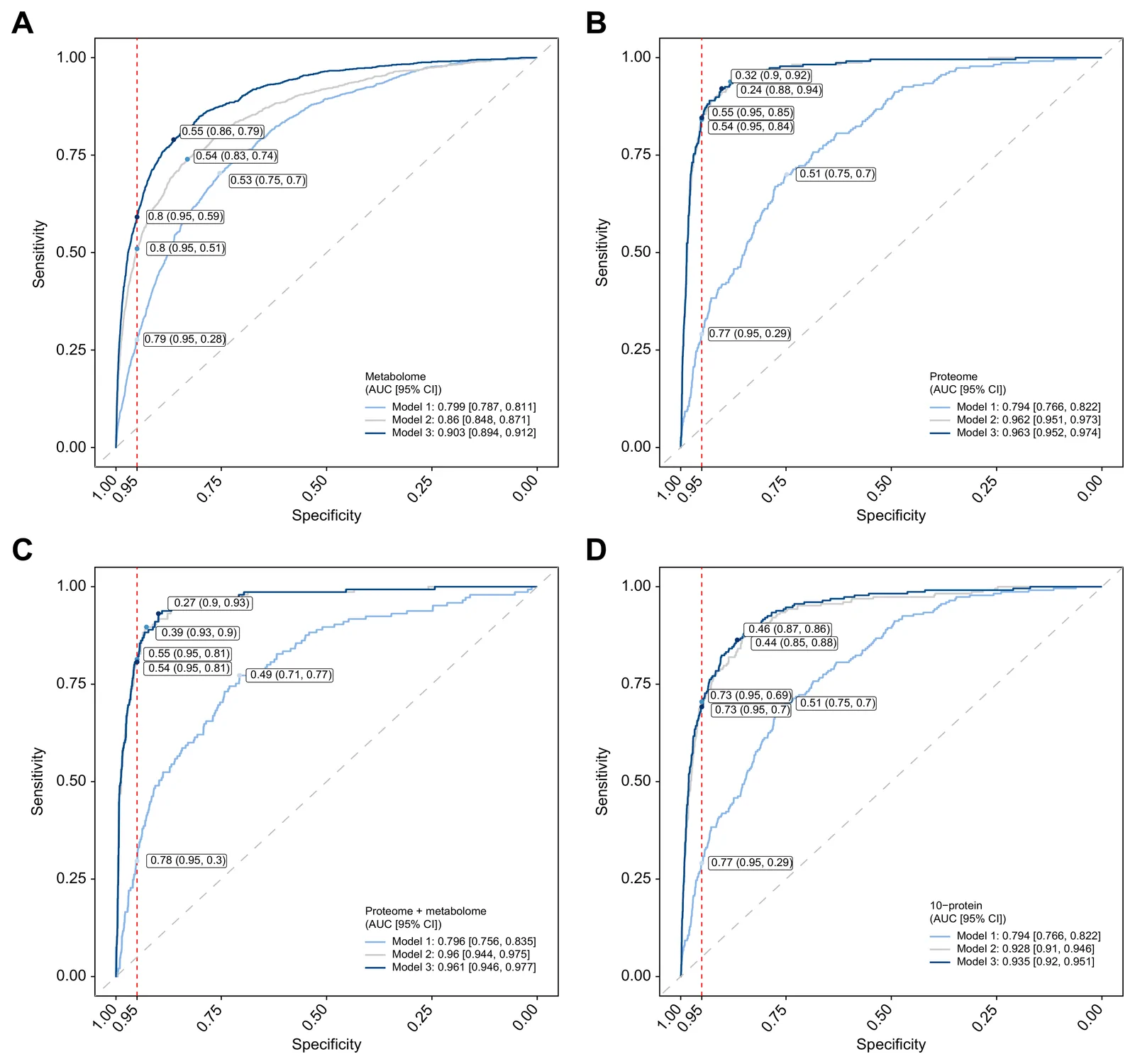
Classification performance of models in differentiating ALD and MASLD. (A) Metabolome-related models; (B) proteome-related models; (C) proteome and metabolome-related models; (D) 10-protein models. Variables of Model 1 include age, sex, BMI, and liver enzymes. Model 2 includes metabolites and/or proteins. Model 3 combines the variables in Model 1 and Model 2. The plots display two thresholds for each model: one for optimal specificity and sensitivity, and another for specificity set at 0.95, followed by corresponding specificity and sensitivity values. ALD, alcohol-related liver disease; MASLD, metabolic dysfunction-associated steatotic liver disease. (This figure appears in color on the web.)

Next, we developed classification models to differentiate ALD and MASLD with the significantly different metabolites and/or proteins selected as above. We compared the classification performance of various models. These included a traditional risk factor model (Model 1) with age, sex, BMI, and liver enzymes (ALT, AST, GGT, and AST/ALT ratio), a molecule-only model (Model 2), and combined models of traditional risk factors and molecules (Model 3). Fig. 3A shows that the metabolite-only model achieved an AUC (95% CI) of 0.86 (0.85–0.87), which increased to 0.90 (0.89–0.91) when traditional risk factors were included. When the specificity of the metabolite-only model was set at 0.95, the sensitivity was only 0.51. It increased to 0.59 when traditional risk factors were included. Additionally, the protein-only model achieved an AUC of 0.96 (0.95–0.97), with the best specificity at 0.88 and sensitivity at 0.94. Its sensitivity was as high as 0.84 with a specificity of 0.95. The model performance did not improve significantly after including additional traditional risk factors or metabolome data (Fig. 3B,C).

Reducing the number of proteins using forward selection, we obtained a model with 13 top proteins (MAMDC4, SSC4D, CEACAM16, CHI3L1, GGT1, C4BPB, CDHR5, OXT, FCAMR, VASN, ADGRD1, PTPRS, and ADAM22). It yielded an AUC (95% CI) plateau of 0.93 (0.91–0.95). The subsequent permutation test showed that a 10-protein concise model (MAMDC4, SSC4D, CEACAM16, CHI3L1, GGT1, C4BPB, CDHR5, OXT, FCAMR and ADAM22) achieved the same AUC of 0.93 (0.91–0.95) as the 13-protein model, with the best specificity at 0.85 and sensitivity at 0.88 (Fig. 3D). Using a specificity cut-off of 0.95 (predicted raw index = 0.73), the sensitivity for detecting ALD was 0.70 in the testing dataset. As a result, 5.0% of patients with MASLD (115 out of 2,302) were classified into the ALD group, while 29.5% of patients with ALD (67 out of 227) were classified into the MASLD group (Fig. S7). The classification performance remained similar when we added traditional risk factors to the 10-protein model (AUC = 0.94, 95% CI 0.92–0.95). However, adding 10 proteins to the traditional risk factor model (AUC 0.79, 0.77–0.82) significantly increased the AUC by 17.8%. Among the 10 selected proteins, CEACAM16 also remained in the concise model built from the PDFF-based diagnosis (Supplementary Results). Furthermore, the classification performance of the models, particularly the proteome models, was not significantly affected by the method used to define liver steatosis (Figs. S8–9; Supplementary Results), potential recall bias from self-reported alcohol intake (Fig. S10), the status of liver steatosis in ALD (Figs. S11–12), ethnicity (Fig. S13), or the imputation of missing values (Figs. S14–15).

Each of the 10 proteins showed a significantly different distribution among ALD, MetALD, and MASLD (Fig. S16), and was significantly associated with alcohol consumption (Table S9). We further performed functional enrichment analysis to assess the expression of the encoding genes of the proteins in relevant tissues and pathways.^18^ Fig. S17 demonstrates that liver tissue is the most significant site for the 10 proteins’ upregulated differentially expressed genes, with C4BPB, CDHR5, CHI3L1, GGT1, MAMDC4, and OXT showing high expression levels in the liver. However, we did not identify any enriched pathways among the 10 proteins.

### Subgroups of MetALD by the classification model

The 10-protein model was further used to classify patients with MetALD into the MASLD group (n = 2,268, 68.8%), influenced mainly by cardiometabolic factors (referred to as cardiometabolic-predominant), or the ALD group (n = 1,030, 31.2%), primarily determined by alcohol (referred to as alcohol-predominant; Fig. S7), using a specificity cut-off of 0.95. Moreover, the concise model based on the PDFF-based diagnosis classified 71.1% (108/152) of patients consistently with the FLI-based diagnosis, with 21 patients in the alcohol-predominant group and 87 in the cardiometabolic-predominant group.

**Fig. 4:**
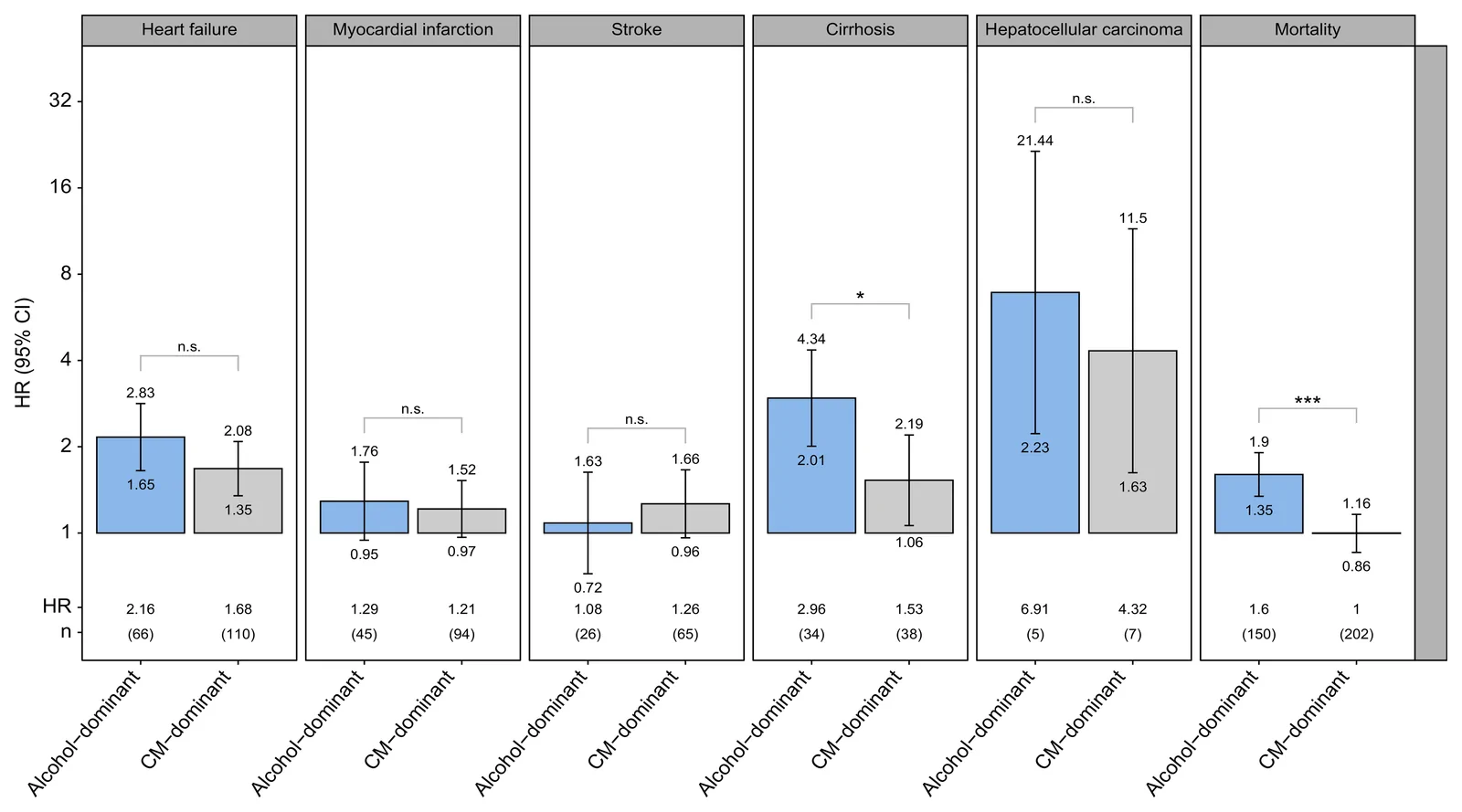
HR of major outcomes in MetALD subgroups, compared to non-SLD controls. Cox regression was performed with adjustments for age, sex, and fasting time. The HR values are positioned beneath the bar plots, accompanied by 95% CI adjacent to the error bars. Number of incident outcome events in each case group is denoted by “n” in the brackets. *** *p* <0.0001, ** *p* <0.001, * *p* <0.0083, # *p* <0.05, n.s. *p* ≥0.05. CM, cardiometabolic; HR, hazard ratio; MetALD, metabolic dysfunction- and alcohol-associated liver disease; SLD, steatotic liver disease.

Patients with MetALD in either subgroup have significantly higher risks of incident heart failure, cirrhosis and hepatocellular carcinoma compared to non-SLD controls (Figs. 4 and S18). When compared with the cardiometabolic-predominant group, the alcohol-predominant group had significantly higher risks of mortality, and cirrhosis (*p* <8.3×10^-3^, Fig. 4). The differences for heart failure and hepatocellular carcinoma between the two MetALD subgroups were not statistically significant. No significant differences were detected for myocardial infarction and stroke between the subgroups or either subgroup and non-SLD controls. Overall, the results remained consistent after accounting for the competing risk of mortality (Fig. S19), excluding participants with any prevalent major outcomes (Fig. S20), and in the advanced model with additional confounding adjustments (Fig. S21).

**Fig. 5:**
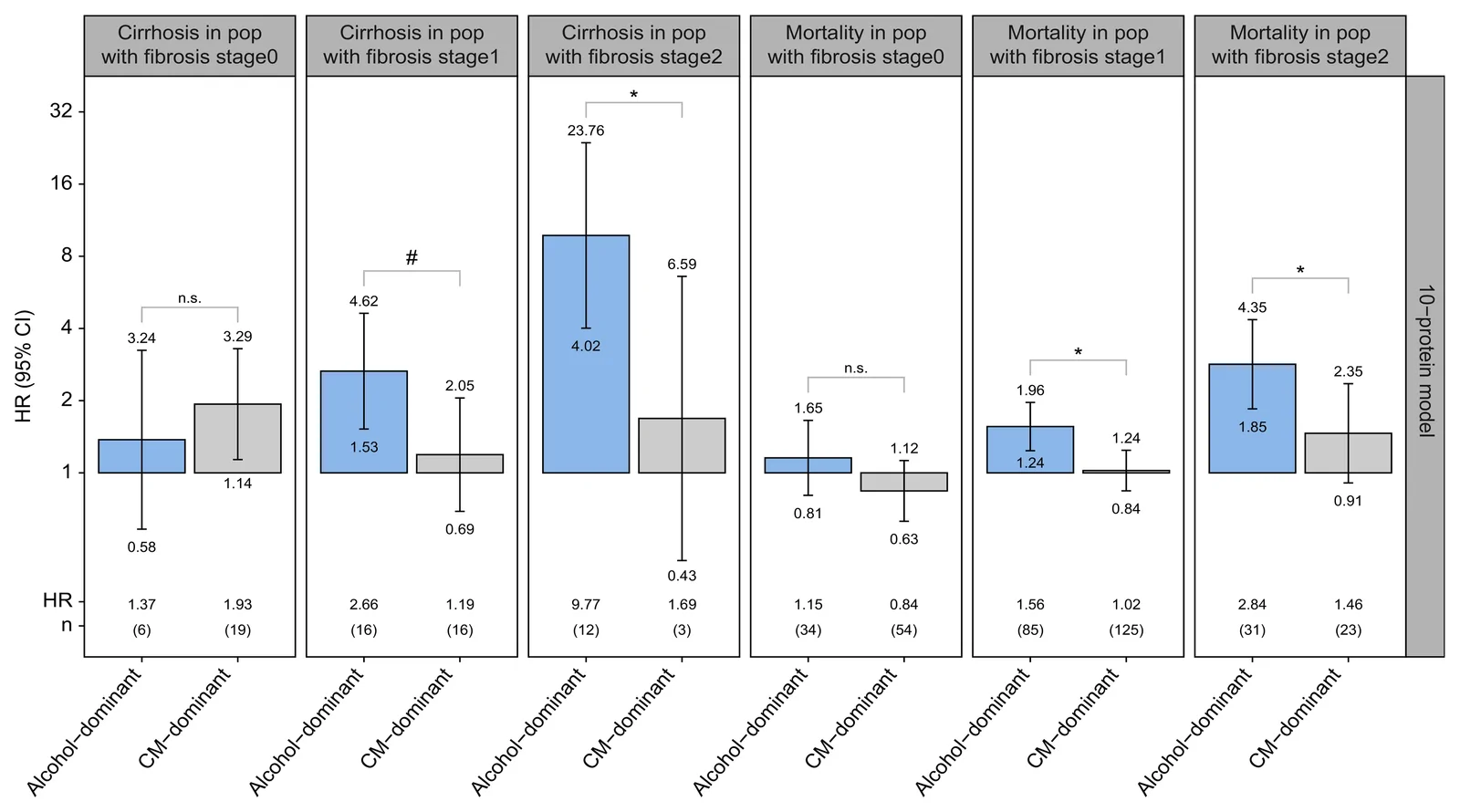
HR of cirrhosis and mortality in MetALD subgroups, compared to non-SLD controls, stratified by fibrosis stages. Cox regression was performed with adjustments for age, sex, and fasting time. The HR values are positioned beneath the bar plots, accompanied by 95% CIs adjacent to the error bars. Number of incident outcome events in each case group is denoted by “n” in the brackets. ***p <0.0001, **p <0.001, *p <0.025, #*p* <0.05, n.s. *p* ≥0.05. pop: population; CM, cardiometabolic; HR, hazard ratio; MetALD, metabolic dysfunction- and alcohol-associated liver disease; SLD, steatotic liver disease.

As liver fibrosis status is a well-established predictor for major outcomes in patients with MASLD, we compared FIB-4, NFS levels and fibrosis stages between the two MetALD subgroups. We found that patients with MetALD in the alcohol-predominant group had significantly higher FIB-4 levels (odds ratio 1.48, 95% CI 1.27–1.73, *p* = 5.0×10^-7^) and a higher prevalence of fibrosis stage 2 (odds ratio 2.01, 95% CI 1.32–3.06, *p* = 1.2×10^-3^) even in the advanced model with more adjustment (Table S10). No significant differences were found for NFS and the prevalence of fibrosis stage 1 between the two MetALD subgroups. We further compared the risk of cirrhosis and mortality, which showed significant differences between MetALD subgroups, across fibrosis stages within the subgroups (Figs. 5 and S22). Although the number of events in each group was small, we still observed a significantly higher risk of cirrhosis and mortality in the alcohol-predominant group compared to the cardiometabolic-predominant group, regardless of whether advanced adjustment was applied. This was true for fibrosis stage 2 and fibrosis stage 1 (*p* <0.025). No significant difference was detected in the population with fibrosis stage 0.

Moreover, we examined the associations of the 10 proteins in the concise model with mortality, incidence of cirrhosis, fibrosis scores and stages, as well as stratifications by fibrosis stage in patients with MetALD, given that these traits differed significantly between the two MetALD subgroups. Fig. S23 shows that all traits, except for incident cirrhosis at fibrosis stage 1, were significantly associated with at least one protein. CHI3L1 showed the strongest and most consistent association with these traits, followed by GGT1, CEACAM16, and SSC4D.

## Discussion

In this study, we distinguished patients with MetALD driven by alcohol-related factors and those driven by cardiometabolic characteristics, using a classification model derived from significant differences in protein profiles between ALD and MASLD. Specifically, the alcohol-predominant MetALD group, identified by 10 proteins (MAMDC4, SSC4D, CEACAM16, CHI3L1, GGT1, C4BPB, CDHR5, OXT, FCAMR, and ADAM22), had significantly higher risks of mortality, and cirrhosis, along with elevated FIB-4 levels and higher fibrosis stages, compared to the cardiometabolic-predominant group. This highlights the value of protein data in disease stratification and its potential to capture the impact of alcohol on MetALD outcomes.

When comparing the risk of major complications, patients with MetALD showed a prognosis similar to MASLD for mortality, stroke, cirrhosis, and hepatocellular carcinoma, but aligned more with ALD for myocardial infarction. This indicates that metabolic and alcohol-related factors could lead to diverse MetALD populations, which underscores the importance of precise MetALD categorisation for guiding interventions and patient stratification. Detailed molecular analysis, as performed here, can assist in the characterization of MetALD and its subtypes.

The models developed in this study, especially the concise model using 10 proteins with an AUC of 0.93, highlight the effectiveness of proteome data in distinguishing ALD and MASLD. This emphasizes the potential of proteomic profiling in capturing distinct molecular differences between steatotic liver conditions, making it a practical clinical tool. Moreover, adding clinical or metabolome data to the protein-only model does not improve classification performance. This indicates that proteome data can effectively capture disease differences contributed by traditional risk factors and metabolome data when differentiating ALD and MASLD. This conclusion was consistently supported across various sensitivity analyses.

The 10 proteins included in the concise model are also significantly associated with alcohol consumption. However, most of their direct associations with alcohol or MASLD remain underexplored, except for GGT1 and OXT. GGT1 (gamma-glutamyltransferase 1) is a well-established indicator of liver injury related to excessive alcohol consumption, while OXT (oxytocin) has shown efficacy in reducing alcohol use disorder effects.^19^ MAMDC4 (MAM domain containing 4), with an AUC of 0.82, is the top differentiating protein; its paralog, MALRD1, regulates bile acid metabolism and hepatic steatosis.^20^ SSC4D (scavenger receptor cysteine rich family member with 4 domains) has been identified as a biomarker for hepatic steatosis in Prader-Willi syndrome.^21^ *CEACAM16* (CEA cell adhesion molecule 16) is genome-wide significantly associated with the interaction between LDL cholesterol and alcohol consumption status.^22^ CHI3L1 (chitinase 3 like 1) is implicated in liver injury and fibrosis due to alcohol.^23^ *C4BPB* (complement component 4 binding protein beta) is mainly expressed in the liver and linked to acute hepatic conditions.^24^ C4BPB protein alone discriminates alcohol-associated hepatitis from healthy controls with an AUC of 0.92.^25^ *CDHR5* (cadherin-related family member 5) is highly expressed in the liver and the protein is a potential hepatocellular carcinoma biomarker.^26^ *FCAMR* (Fc alpha and mu receptor) expression has been reported to help distinguish MASLD and non-MASLD.^27^ ADAM22 (ADAM metallopeptidase domain 22) is associated with AST/ALT ratio.^28^ We did not identify enriched pathways from the 10 proteins, likely due to their small number and limited statistical power. It may also be the case that these proteins, which were detected through the current strategy, reflect different pathways.

The 10-protein model effectively identifies alcohol-predominant patients as having higher risks of mortality and cirrhosis compared to those in the cardiometabolic-predominant group. This observation suggests that the alcohol-predominant group may significantly contribute to mortality and cirrhosis risk among patients with MetALD. These findings imply that proteomics can effectively capture prognostic information regarding the impact of alcohol consumption on MetALD. Moreover, measuring alcohol consumption through questionnaires can be unreliable due to high recall bias in the MetALD group^3^ or reverse causation, where individuals may reduce alcohol intake in response to health issues. Therefore, proteomic profiling may serve as a valuable tool in monitoring the impact of alcohol consumption on MetALD prognosis. Moreover, clinicians can use an instantly available alcohol questionnaire to screen for MetALD alongside the 10 selected proteins to classify MetALD subgroups effectively with the concise protein model and cut-off. This strategy helps identify patients who need more intensive medical interventions, such as personalized lifestyle education or increased follow-ups, to reduce long-term cirrhosis or mortality risk.

We found that the alcohol-predominant MetALD group had significantly higher FIB-4 levels and more advanced fibrosis stages than the cardiometabolic-predominant group, which indicates severer liver disease progression in the alcohol-predominant group. This aligns with our findings regarding the increased risk of hepatic outcomes and mortality among alcohol-predominant patients. Previous studies have also reported strong associations between alcohol-related liver diseases and liver fibrosis,^29^ further corroborating our model’s effectiveness in classifying MetALD. Earlier research has also shown that heavy alcohol consumers with liver fibrosis have a higher risk of progressing to cirrhosis compared to individuals who do not consume alcohol.^29^ However, further investigation into this interaction and its underlying mechanisms is needed.

Our study has several strengths, including a long follow-up period, a large sample size, and the use of advanced omics technologies. Nonetheless, there are limitations to acknowledge. First, we defined hepatic steatosis using FLI, which is less precise than imaging or biopsy, although it is easy to access for clinical screening. Although the PDFF-based diagnosis showed similar model performance to the FLI-based diagnosis, it was based on a limited number of samples. Additionally, the PDFF-based diagnosis included too few incident cases to robustly validate associations with major outcomes. Therefore, further validation with larger PDFF-based cohorts remains necessary. Second, while self-reported alcohol data are convenient for screening clinically and our sensitivity analysis shows the robustness of the classification models, we cannot completely exclude recall bias from the association between MetALD subgroups and major outcomes due to the small sample size in the sensitivity analysis. Third, the UK Biobank is subject to a selection bias related to “healthy volunteers”, who have lower rates of comorbidities than the general population.^30^ This bias may restrict the generalizability of our results to a broader population, particularly regarding the prevalence or incidence rates of SLD and its subtypes, but not for conclusions drawn from exposure-disease relationships.^30^ Fourth, external validation could enhance the scientific rigor of our conclusions, though we have employed a careful design in the current study to avoid overfitting by using a predefined independent testing dataset and an independent non-European population in the UK Biobank for validation. Fifth, we observed no increased risk of mortality in patients with cardiometabolic-predominant MetALD compared to non-SLD controls, despite elevated risks of heart failure, cirrhosis, and hepatocellular carcinoma. This may be due to the heterogeneous causes of death but warrants further investigation.

In conclusion, our findings highlight the importance of precise classification for personalized medicine in MetALD. Molecular profiles, especially protein profiles, are valuable for categorising MetALD subtypes and developing diagnostic and prognostic tools. Future studies should focus on validating models in large liver imaging-based cohorts and exploring the comprehensive clinical and pathophysiological characteristics of these MetALD subgroups.

## Supplementary Material

Supplementary data to this article can be found online at https://doi.org/10.1016/j.jhep.2025.05.026.

Multimedia component 2

Multimedia component 3

Supplementary Methods

Supplementary Tables

## Figures and Tables

**Table 1:** Characteristics of the study population.

Variables	Non-SLD (n = 217,740)	MASLD (n = 124,034)	MetALD (n = 34,147)	ALD (n = 11,220)
Age (years)	56.42 ± 8.21	57.73 ± 7.75	57.17 ± 7.69	56.47 ± 7.67
Male,	70,404 (32)	72,371 (58)	25,743 (75)	10,036 (89)
BMI (kg/m^2^)	24.89 ± 2.86	31.77 ± 4.55	30.64 ± 3.85	30.28 ± 3.99
Waist circumference (cm)	82.29 ± 8.94	102.64 ±10.18	102.15 ± 9.34	103.26 ± 9.88
Education				
College or University degree	77,045 (35)	32,075 (26)	9,837 (29)	2,779 (25)
A levels, AS levels or equivalent	25,760 (12)	12,713 (10)	3,726 (11)	1,211 (11)
CSEs or equivalent	11,126 (5)	7,320 (6)	2,053 (6)	739 (7)
NVQ or HND or HNC or equivalent	11,776 (5)	10,143 (8)	3,028 (9)	1,111 (10)
O levels GCSEs or equivalent	47,404 (22)	26,617 (21)	7,359 (22)	2,500 (22)
Other professional qualifications	11,243 (5)	6,933 (6)	1,761 (5)	554 (5)
None	33,386 (15)	28,233 (23)	6,383 (19)	2,326 (21)
Physical activity				
High	93,888 (43)	42,013 (34)	12,659 (37)	4,383 (39)
Moderate	89,704 (41)	50,917 (41)	13,997 (41)	4,340 (39)
Low	34,148 (16)	31,104 (25)	7,491 (22)	2,497 (22)
Smoking status				
Never	136,041 (62)	64,592 (52)	12,425 (36)	3,123 (28)
Previous	63,803 (29)	47,251 (38)	17,017 (50)	5,636 (50)
Current	17,896 (8)	12,191 (10)	4,705 (14)	2,461 (22)
Pack years^†^,	6.5 (0.03, 19.88)	13.6 (0.03, 29.5)	14.25 (0.03, 29.62)	20.6 (4.5, 37.5)
Weekly alcohol grams^‡^,	76.8 (48, 112)	95.2 (60, 138.4)	264 (224.8, 320)	532 (464, 642.4)
AST (U/L)	23.2 (20.2, 27)	25.9 (22.1, 30.8)	27.1 (23.2, 32.7)	29.5 (24.8, 36.9)
ALT (U/L)	17.45 (13.95, 22.27)	25.42 (19.43, 34.19)	27.54 (21.1, 36.72)	29.85 (22.52, 40.77)
AST/ALT ratio	1.38 ± 0.42	1.07 ± 0.35	1.04 ± 0.34	1.07 ± 0.37
GGT (U/L)	20.3 (15.8, 27.8)	36.3 (26.2, 54.3)	46.8 (33, 71.2)	66.25 (44.2, 108.5)
Systolic blood pressure (mmHg)	134.45 ± 18.68	141.76 ± 17.53	144.48 ± 17.38	147.88 ± 17.71
Diastolic blood pressure (mmHg)	79.61 ± 9.71	85.05 ± 9.73	86.8 ± 9.65	88.55 ± 10.02
Hypertension	91,802 (42)	84,657 (68)	24,457 (72)	8,697 (78)
Triglycerides (mmol/L)	1.22 (0.92, 1.65)	2.14 (1.6, 2.91)	2.13 (1.56, 2.93)	2.18 (1.57, 3.1)
HDL-cholesterol (mmol/L)	1.55 ± 0.36	1.22 ± 0.28	1.33 ± 0.3	1.41 ± 0.34
Lipid-lowering drugs	33,830 (16)	37,528 (30)	10,090 (30)	3,217 (29)
Glucose (mmol/L)	4.95 ± 0.74	5.28 ±1.17	5.23 ± 1	5.3 ± 1.02
HbA1c (mmol/mol)	34.91 ± 4.41	37.89 ± 7.08	36.51 ± 5.97	36.46 ± 6.13
Type 2 diabetes	4,429 (2)	14,308 (12)	2,561 (7)	926 (8)
CRP (mg/L)	0.98 (0.51, 1.96)	2.23 (1.19, 4.3)	1.91 (1.04, 3.62)	1.92 (1.05, 3.63)
PDFF (%)	2.6 (2, 3.8)	5.2 (3.3, 9.7)	5.7 (3.5, 9.9)	6.6 (3.94, 11.67)
FLI	26.97 ± 16.79	80.98 ±11.57	81.21 ±11.38	83.98 ±11.36
FIB-4	1.26 (0.99, 1.58)	1.2 (0.94, 1.52)	1.24 (0.97, 1.57)	1.32 (1.03, 1.72)
NFS	-2.03 ± 1.06	-1.43 ± 1.22	-1.6 ± 1.15	-1.54 ± 1.21
Fibrosis stage				
Fibrosis stage 0	109,702 (50)	52,009 (42)	15,184 (44)	4,434 (40)
Fibrosis stage 1	102,987 (47)	64,955 (52)	17,388 (51)	5,933 (53)
Fibrosis stage 2	5,051 (2)	7,070 (6)	1,575 (5)	853 (8)

ALT, alanine aminotransferase; AS, Advanced Subsidiary; AST, aspartate aminotransferase; CES, Certificate of Secondary Education; CRP, C-reactive protein; FIB-4, fibrosis-4 index; FLI, fatty liver index; GCSE, General Certificate of Secondary Education; GGT, gamma-glutamyltransferase; HbA1c, haemoglobin A1c; HNC, Higher National Certificates; HND, Higher National Diploma; MASLD, metabolic dysfunction-associated steatotic liver disease; MetALD, metabolic dysfunction- and alcohol-associated liver disease; (MRI-) PDFF, MRI-estimated proton density fat fraction; NFS, NAFLD fibrosis score; NVQ, National Vocational Qualification; SLD, steatotic liver disease.

Data are presented as n (%), or median (IQR) or mean ±SD.

† Never smokers were not considered when calculating pack-years.

‡ Never drinkers were not considered when calculating weekly alcohol grams.

## Data Availability

UK Biobank data are publicly available to bona fide researchers upon application at http://www.ukbiobank.ac.uk/using-the-resource/.

## Highlights

- A 10-protein model achieves an AUC of 0.93 for distinguishing ALD from MASLD.
- Protein-only model captures the contribution of clinical factors and metabolites in differentiating ALD and MASLD.
- Alcohol-dominant MetALD linked to higher risks of mortality, cirrhosis, and fibrosis than cardiometabolic-dominant MetALD.
- Subtyping MetALD using the proteome can improve prognostication in patients with MetALD.

## Impact and implications

This study underscores the critical importance of distinguishing subtypes of metabolic dysfunction- and alcohol-associated liver disease (MetALD) using proteomic data, providing a foundation for personalized treatment strategies. The findings hold significant relevance for healthcare providers, researchers, and policymakers by highlighting the differing risks associated with alcohol-predominant vs. cardiometabolic-predominant MetALD. Clinicians can apply the classification model developed in this study to more accurately assess patients and guide targeted therapies and preventive measures based on individual profiles. However, limitations of the study, such as reliance on self-reported alcohol consumption and the specificity of diagnostic criteria, necessitate further validation in diverse cohorts.

## Abbreviations

ALD: alcohol-related liver disease
ALT: alanine aminotransferase
AST: aspartate aminotransferase
FIB-4: fibrosis-4 index
FLI: fatty liver index
GGT: gamma-glutamyltransferase
MASLD: metabolic dysfunction-associated steatotic liver disease
MetALD: metabolic dysfunction- and alcohol-associated liver disease
(MRI-)PDFF: MRI-estimated proton density fat fraction
NFS: NAFLD fibrosis score
SLD: steatotic liver disease
