# Supplementary material for: Dissecting metabolic dysfunction- and alcohol-associated liver disease (MetALD) using proteomic and metabolomic profiles
Source: J Hepatol. Author manuscript; Available in PMC 2026 Sep 4. (PMC7619437; doi:10.1016/j.jhep.2025.05.026)
Supplement: Supplementary file 2 [file EMS217801-supplement-Supplementary_Methods.pdf]

Dissecting metabolic dysfunction- and alcohol-associated liver disease (MetALD) using proteomic and metabolomic profiles

Jun Liu, Sihao Xiao, Sile Hu, Rui Huang, Lingyan Chen, Jeremy W Tomlinson, Jeremy F Cobbold, Joanna M M Howson, Cornelia M van Duijn

Table of contents

Supplementary Methods……………………………………………………...…2
Supplementary Results………………………………………………………..…3
Supplementary Figures……………………………………………………….5
Supplementary Tables……………………………………………………….28
Supplementary References…………………………………………...……29

Supplementary Methods

Missing value imputation

For variables used to define FLI and the five cardiometabolic criteria for MASLD, their missing rates were all less than 20%. Fast imputation of missing values was performed in the UK Biobank population through the R package *missRanger*.[1–3] The imputation used baseline information, included age, sex, smoking status, pack-years of smoking, alcohol intake frequency, physical activity from International Physical Activity Questionnaire (IPAQ) groups, ethnicity, BMI, education, blood pressure and waist-hip ratio. In summary, the large matrix was imputed with a maximum of ten chained interactions and 200 trees, weighted by the number of non-missing values, with three candidate non-missing values selected from the predictive mean matching steps.

Sensitivity analyses for the classification models

We conducted sensitivity analyses to test the robustness of our classification models. We compared the results between the PDFF-based and FLI-based diagnosis and validated the FLI-based models within PDFF diagnosed disease categories (details below). To address potential recall bias from self-reported alcohol intake, we assessed the model performance in two extreme groups less likely to be biased: patients with MASLD who reported never drinker and ALD patients with prevalent alcohol-related disorders based on self-reports or ICD codes from hospital records (Supplementary Table 1). To examine if excluding ALD patients without liver steatosis could bias our findings, we evaluated model performance in two scenarios: 1) including ALD patients without steatosis in the initial testing dataset, thus comparing MASLD patients with ALD patients irrespective of steatosis status; and 2) comparing MASLD patients in the testing dataset with ALD patients without liver steatosis. Besides validating the models in the predefined testing dataset as above, we also validated the European-based models in the non-European population from the UK Biobank. Additionally, we compared the model performance between non-imputed and imputed populations to assess potential bias from missing values imputation.

Supplementary Results

Details of the sensitivity analysis based on PDFF-defined approach

To investigate whether the use of FLI for diagnosing liver steatosis introduces biases into our findings, we incorporated a PDFF-based approach in the sensitivity analysis. In this analysis, we used PDFF ≥ 5 to diagnose liver steatosis (referred to as PDFF-defined diagnosis) instead of the primary FLI-based diagnosis, within a sub-cohort of the UK Biobank that has liver MRI imaging measurements available (n=37,802).[4] We conducted various analyses based on the PDFF-based diagnosis to replicate and/or validate our primary findings. These analyses included: 1) Comparing the PDFF-based diagnosis with the FLI-based diagnosis for clinical profiles and molecular associations among MetALD, MASLD, and ALD. 2) Validating the classification models created by FLI-based approach in participants with diseases diagnosed by PDFF. 3) Replicating the modelling strategy for classifying ALD and MASLD using the PDFF diagnosis, including estimating classification models between traditional risk factors and/or molecules, and establishing a protein-only concise model. All validations were performed in the predefined independent testing dataset only to avoid overlapping bias.

With PDFF-based diagnosis, we identified 2,374 (6.3%) participants with MetALD, 7,128 (18.9%) with MASLD, 603 (1.6%) with ALD, and 21,277 (56.3%) participants as non-SLD controls. Although liver MRI imaging was measured 4-10 years after the baseline when FLI was derived, we observed a significant overlap in the defined SLD groups between FLI-based diagnosis and PDFF-based diagnosis. Specifically, 1,941 (81.8%) MetALD patients were also identified as MetALD patients by FLI, 4,310 (60.5%) for MASLD, 433 (71.8%) for ALD, and 17,402 (81.8%) for non-SLD controls.

Supplementary Figure 1 shows that the clinical profiles of SLD subtypes, ALD versus MASLD, and alcohol intake in the PDFF-based and FLI-based analyses are generally consistent. The significant findings from PDFF-based diganosis were also significant in the FLI-based results and in the same direction ($p<2.0\times10^{-3}$; 113 out of 116 significant associations, 97.4%). Due to the small number of incident major outcome cases with available PDFF diagnosis and limited follow-up years (n<5), we were unable to detect significant differences in the risk of major outcomes among SLD subtypes. Nonetheless, we still observed that ALD patients had higher risks of stroke, cirrhosis, and mortality compared to MetALD and MASLD, whose risks align closely with each other (Supplementary Figure 3).

Using PDFF-based diagnosis, we identified 102 metabolites and 32 proteins that were significantly different between ALD patients and MASLD patients. All of these metabolites and proteins were reported as significant in the FLI-based results in the same direction. The correlation of effect estimates of individual associations between the PDFF-based results and the FLI-based results was high, with a correlation coefficient of 0.98 (95%CI: [0.97, 0.98]) for metabolites and 0.72 [0.71, 0.74] for proteins (Supplementary Figure 6).

We then validated the primary classification models generated from the FLI-based diagnosis in the independent testing dataset with patients diagnosed by PDFF. Consistent results were observed (Supplementary Figure 8): high classification performance remains for protein-only model (AUC=0.94); adding age, sex, BMI, liver enzymes, or

metabolome information did not significantly improve its AUC; and the ten-protein concise model showed excellent performance in distinguishing ALD and MASLD (AUC=0.97).

Lastly, we applied the same modelling strategy for the molecular classification of ALD and MASLD using the PDFF-based diagnosis. Although the total sample size for ALD and MASLD represented only 5.7% of that used in the FLI-based diagnosis, our primary conclusions remained significant (Supplementary Figure 9): proteomic profiling effectively distinguished between ALD and MASLD, and incorporating additional clinical or metabolomic data into the proteome-only model did not enhance its classification performance. In contrast to the primary results through FLI-based diagnosis, the classification performance of traditional risk factors was unstable across different diagnosis, and no significant differences were identified between three models for metabolome (Supplementary Figure 9A, $p>0.05$). Additionally, we identified four key proteins (CEACAM16, CCER2, RTN4R, and IL12A/IL12B) in the concise protein model, achieving a high AUC of 0.97 (Supplementary Figure 9D). Among them, CEACAM16 was selected in both the FLI-based approach and the PDFF-based approach, with a highest AUC to distinguish between ALD and MASLD using individual protein (AUC=0.85) in the PDFF-based approach and the third highest AUC (AUC=0.82) in the FLI-based approach. Unfortunately, we were unable to replicate analyses relevant to the risk of major outcomes in MetALD subgroups due to the limited number of incident cases available.

Supplementary Figures

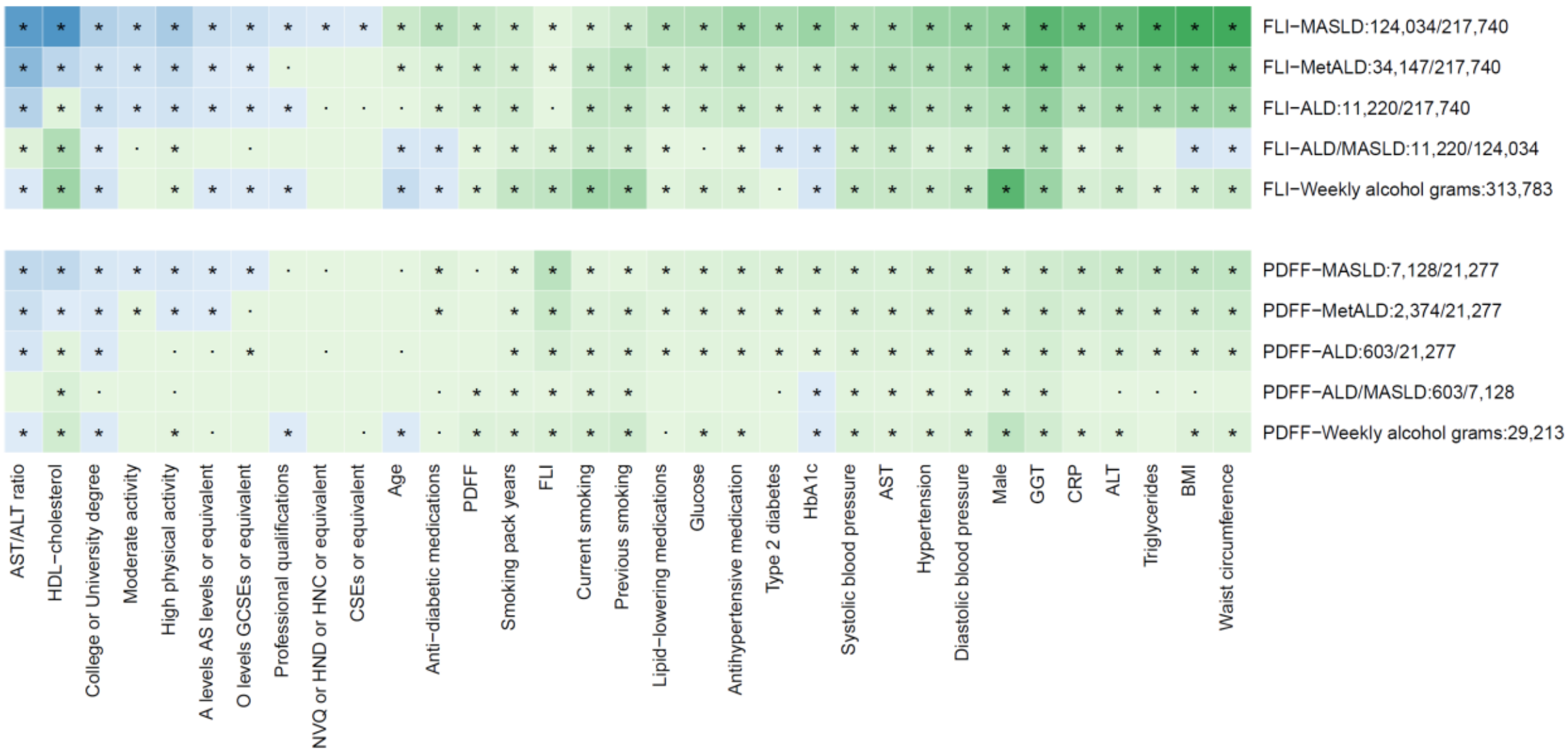

Fig. S1 Comparison of SLD-related clinical profiles among SLD subtypes, ALD versus MASLD, and alcohol intake in FLI-based and PDFF-based diagnoses. Sample sizes for the analyses (cases/controls or total samples) are listed. Logistic/linear regression was performed with adjustment for age, sex, and fasting time. Never drinkers were excluded for the association with weekly alcohol grams. Green indicates positive, blue indicates negative associations, with colour depth reflecting the magnitude of the z-score (range: -/+ 270). Hierarchical clustering was used to group the clinical variables. * $p<2.0\times10^{-3}$, · $p<0.05$.

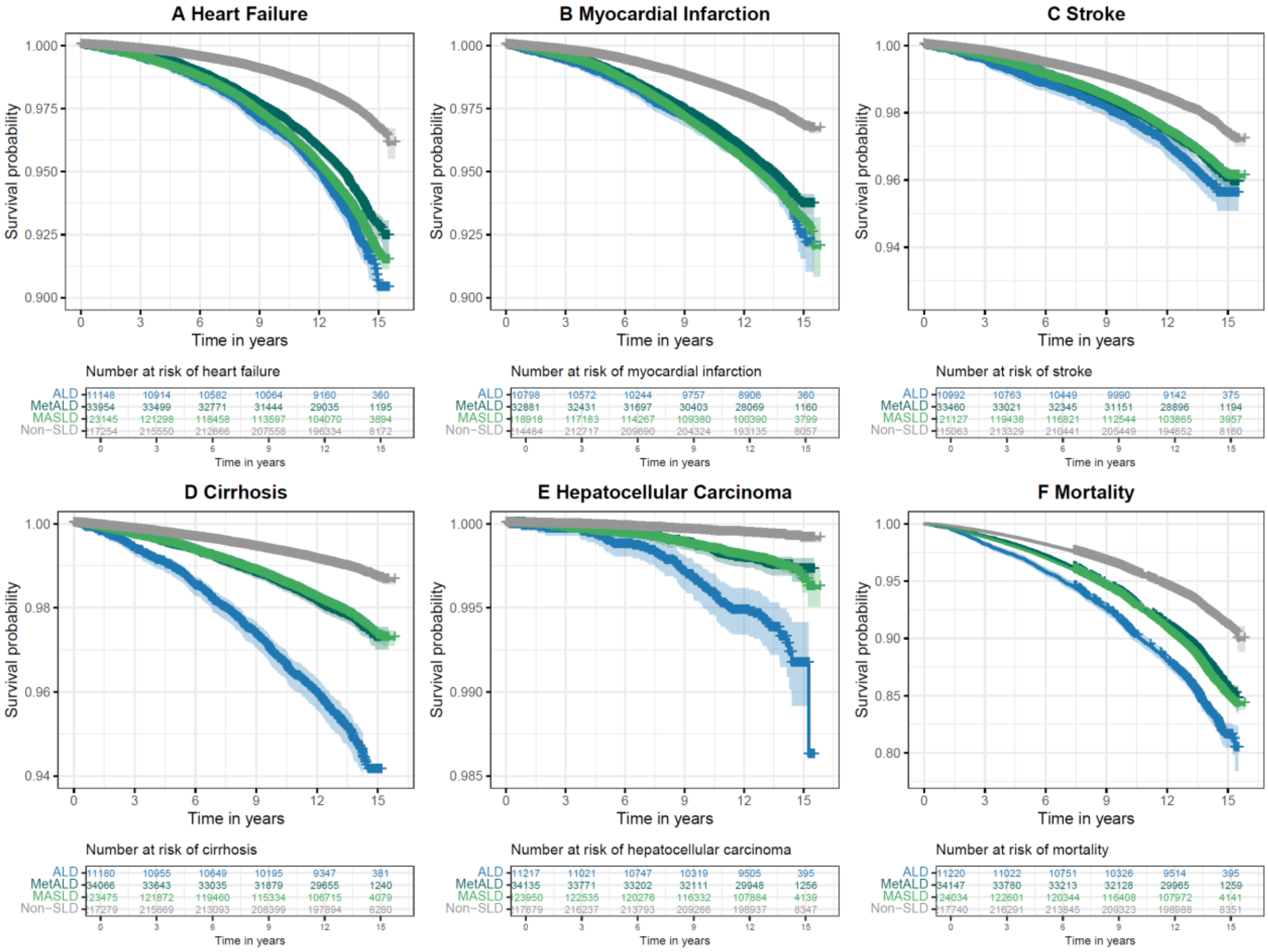

Fig. S2 Survival curves of major outcomes across SLD subtypes and non-SLD controls.

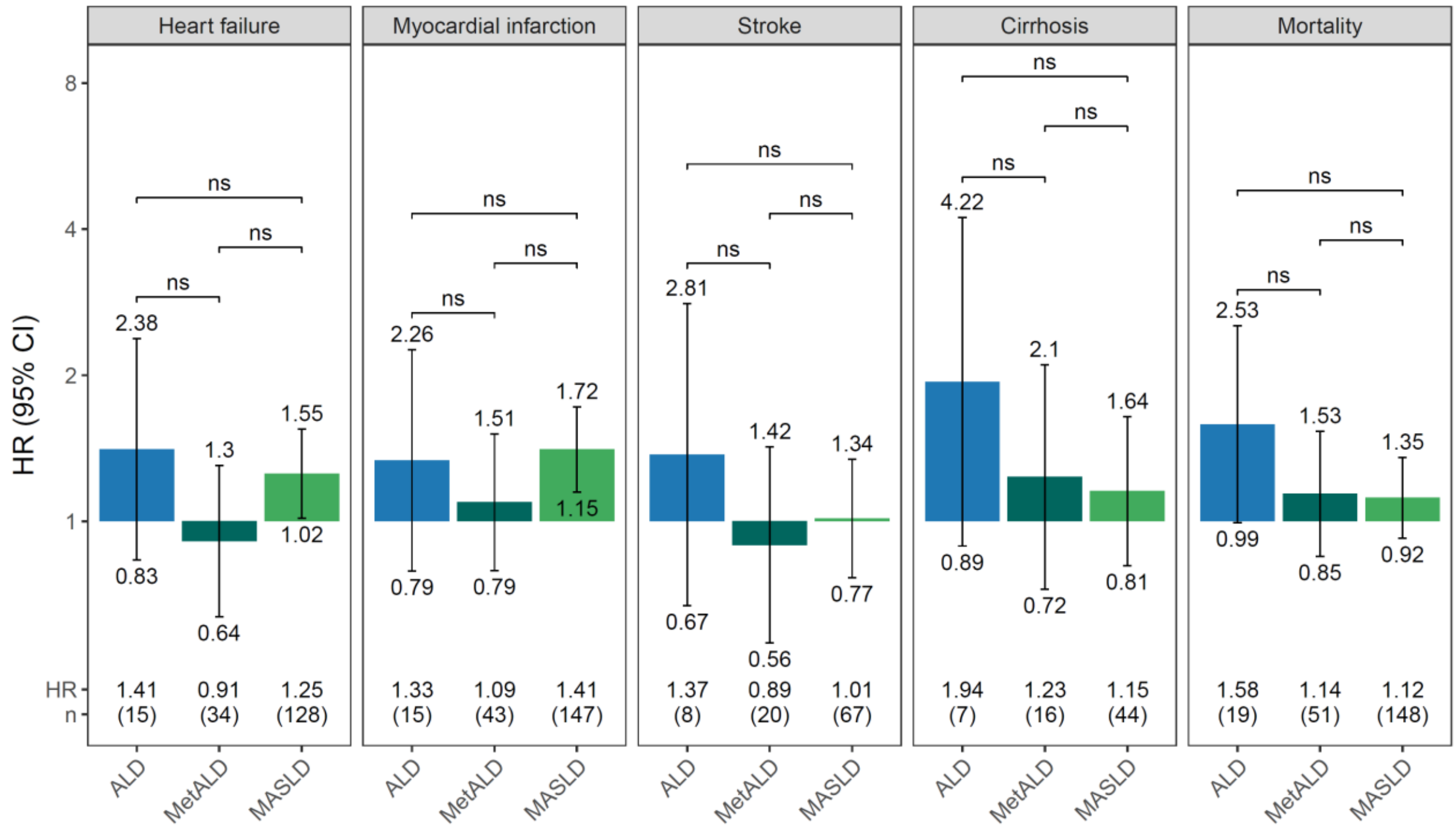

Fig. S3 HR of major outcomes in patients with ALD, MetALD and MASLD, compared to non-SLD controls, using PDFF-based diagnosis. Cox regression was performed with adjustments for age, sex, and fasting time. The HR values are positioned beneath the bar plots, accompanied by 95% CI adjacent to the error bars. Number of incident outcome events in each case group is denoted by "n" in the brackets. *** p<0.0001, ** p<0.001, * $p<2.8\times10^{-3}$, · p<0.05, ns $p\geq0.05$.

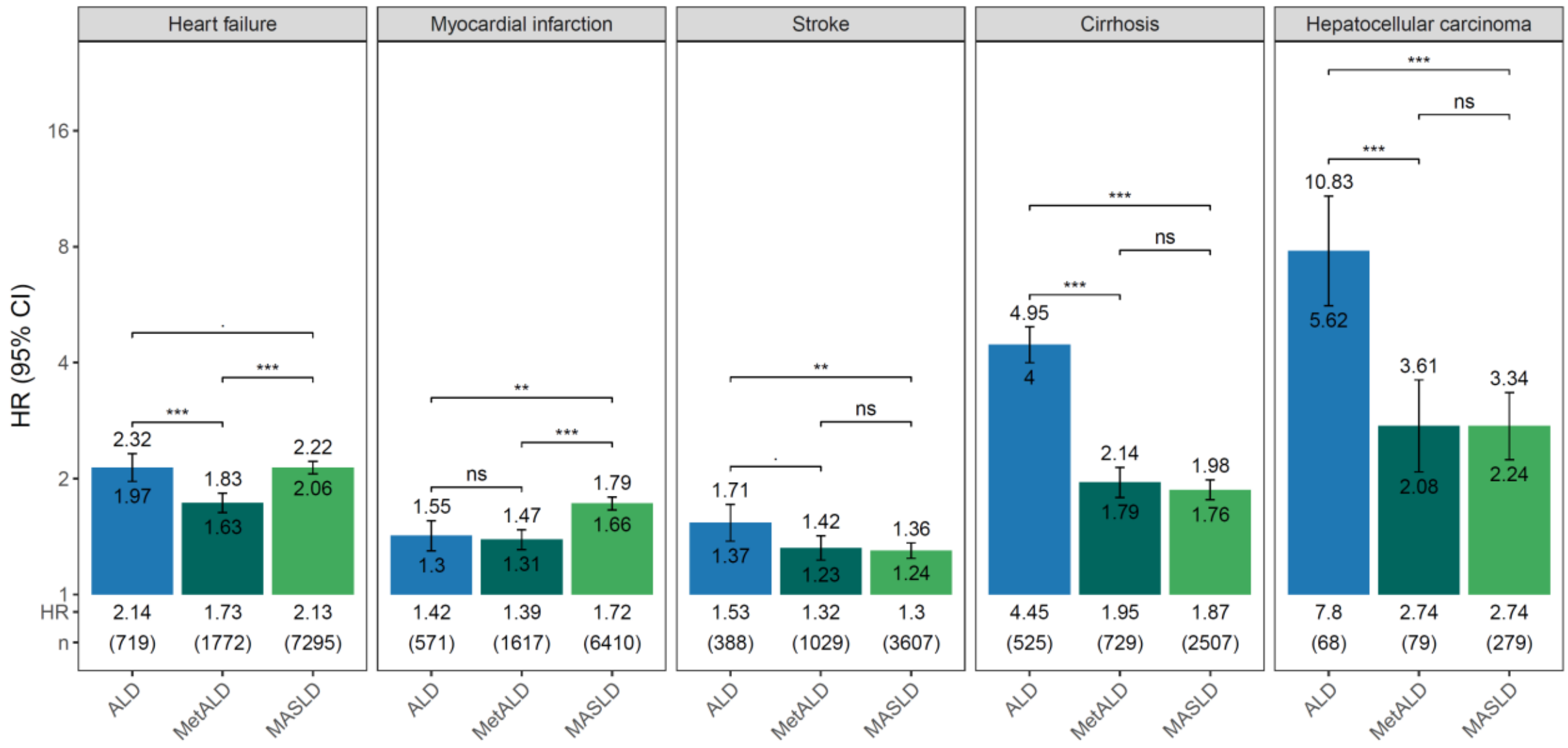

Fig. S4 HR of major outcomes in patients with ALD, MetALD and MASLD, compared to non-SLD controls, accounting for competing risk of mortality. Cox regression was performed with adjustments for age, sex, and fasting time. The HR values are positioned beneath the bar plots, accompanied by 95% CI adjacent to the error bars. Number of incident outcome events in each case group is denoted by "n" in the brackets. *** p<0.0001, ** p<0.001, * $p<2.8\times10^{-3}$, · p<0.05, ns $p\geq0.05$.

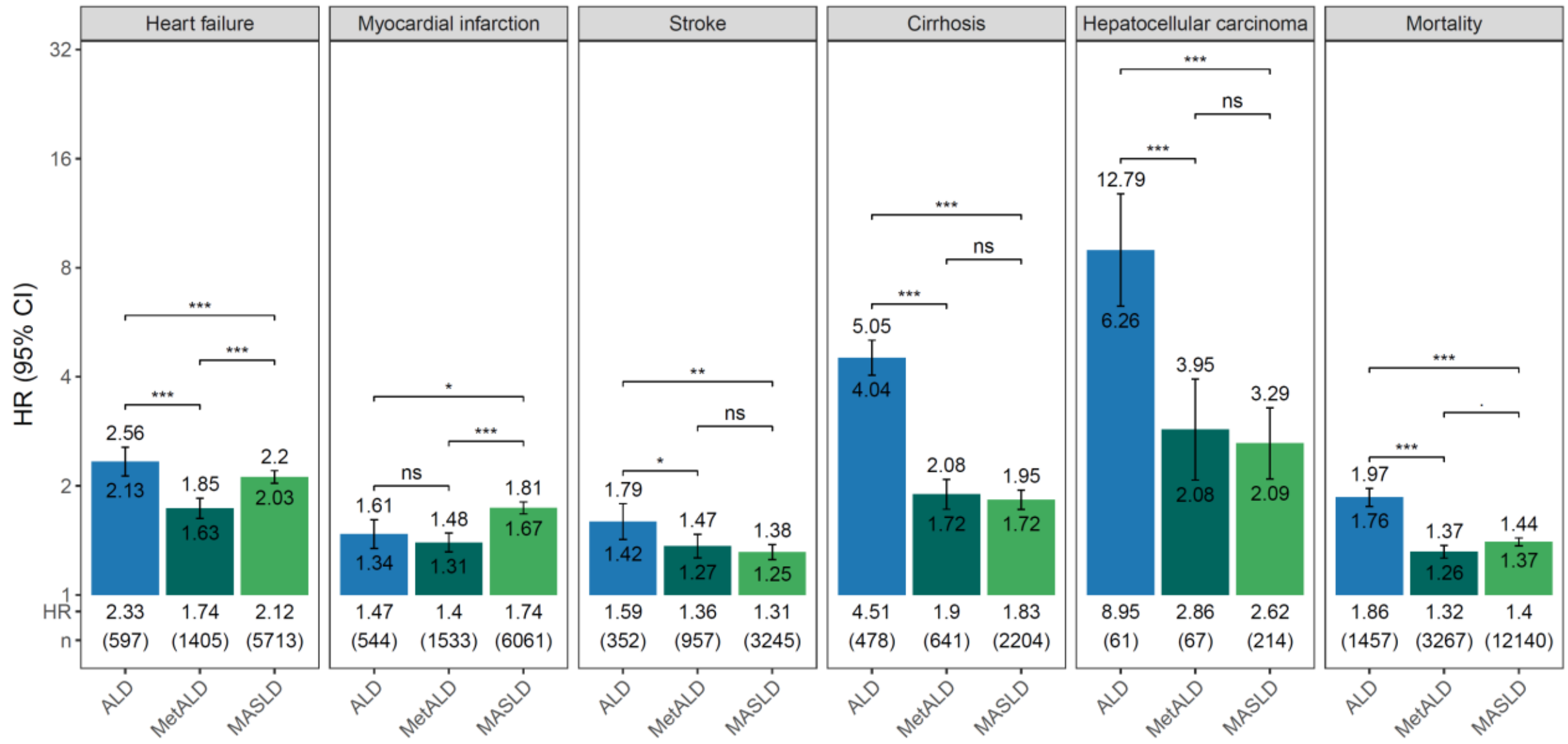

Fig. S5 Sensitivity analysis: HR of major outcomes in patients with ALD, MetALD and MASLD, compared to non-SLD controls. Patients with any major outcomes at baseline were excluded from the survival analysis. Cox regression was performed with adjustments for age, sex, and fasting time. The HR values are positioned beneath the bar plots, accompanied by 95% CI adjacent to the error bars. Number of incident outcome events in each case group is denoted by "n" in the brackets. *** $p<0.0001$, ** $p<0.001$, * $p<2.8\times10^{-3}$, · $p<0.05$, ns $p\geq0.05$.

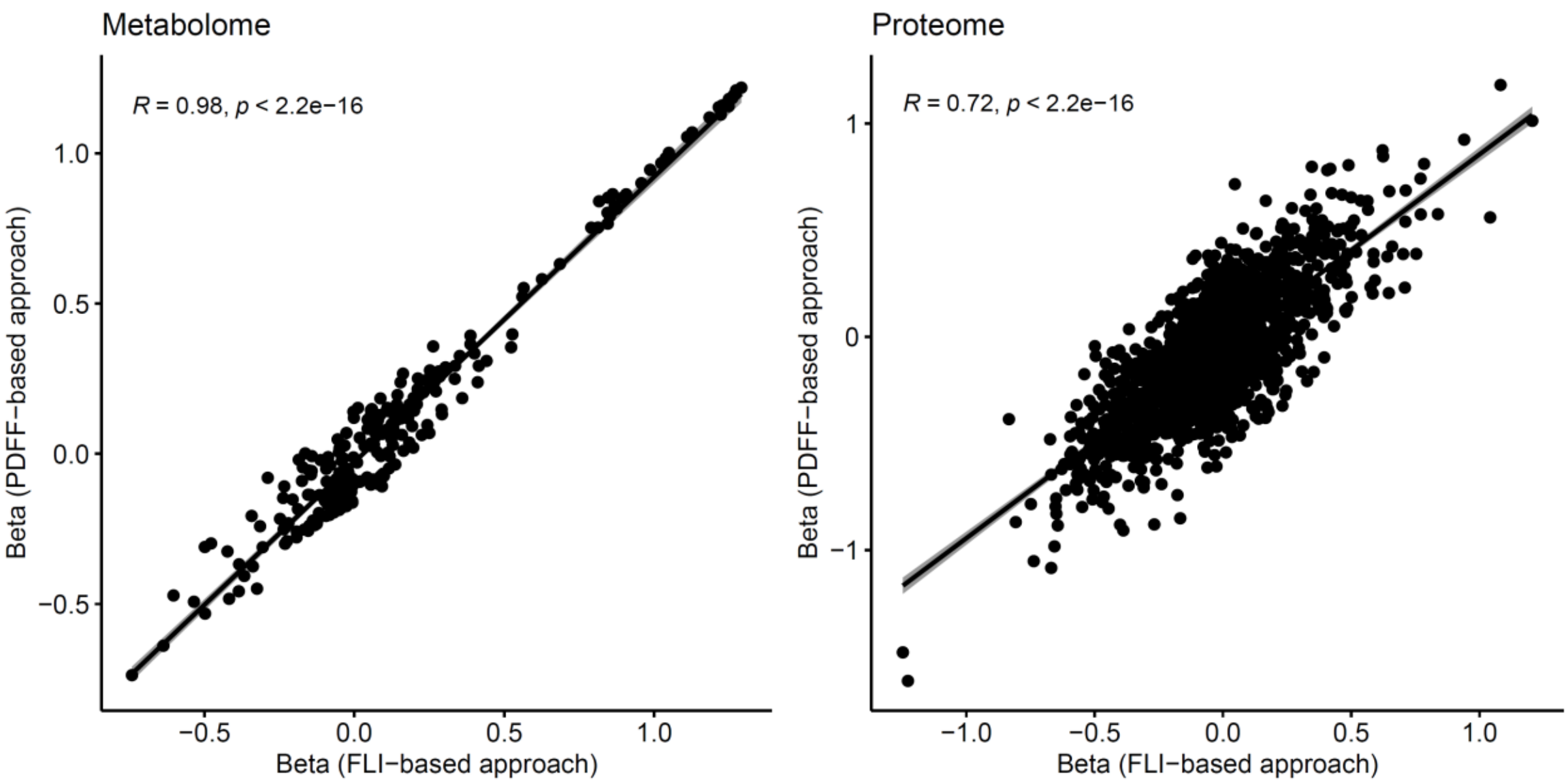

Fig. S6 Correlation of molecules' effect estimates in differentiating ALD and MASLD using PDFF-based and FLI-based approaches. Pearson's correlation was performed for the correlation coefficient.

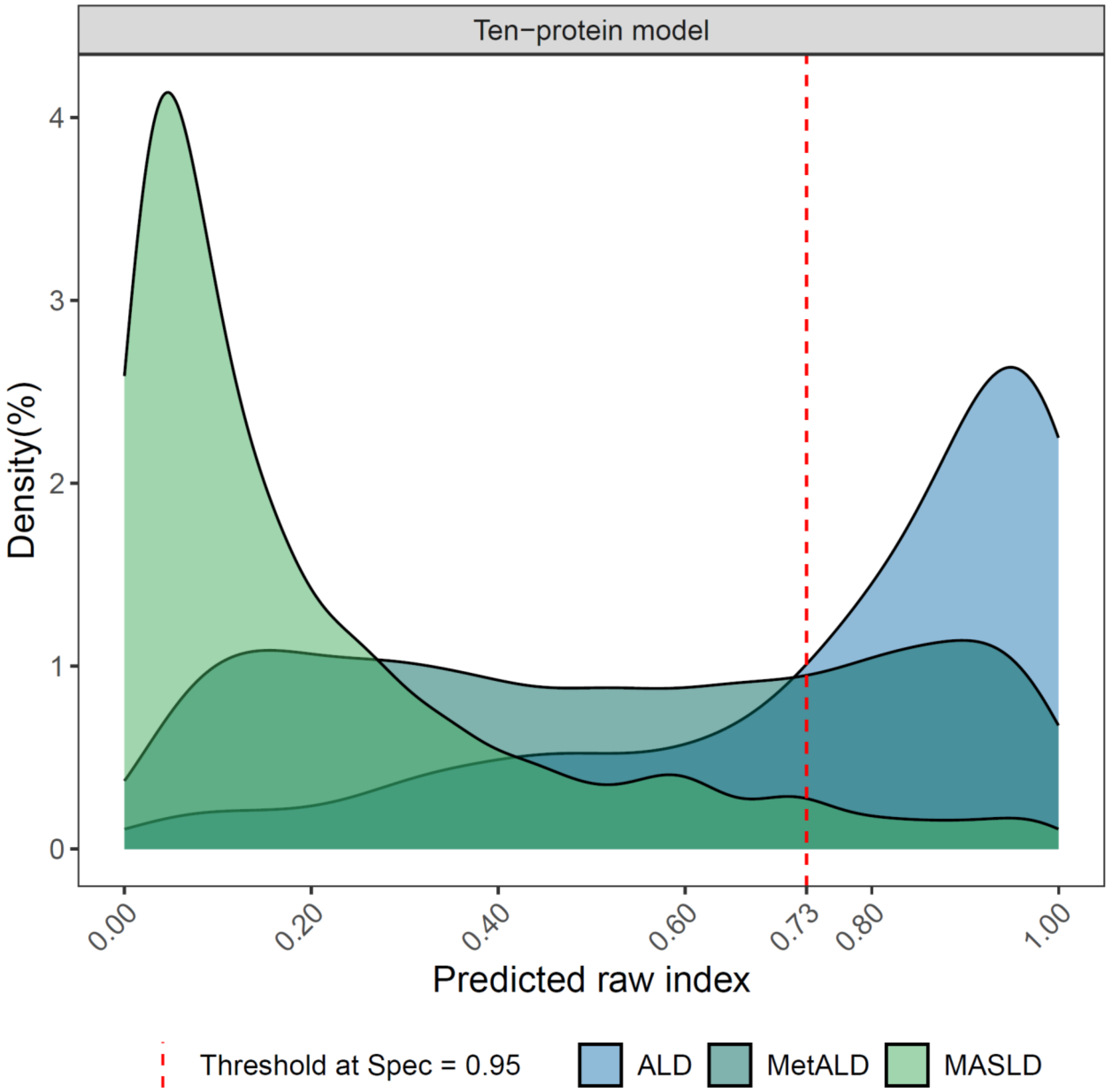

Fig. S7 The probability of ALD calculated from the ten-protein model across ALD, MetALD, and MASLD. The predicted raw index presented for ALD (n=227) and MASLD (n=2,302) was calculated in the testing dataset. The predicted raw index presented for MetALD (n=3,298) was calculated in the MetALD patients.

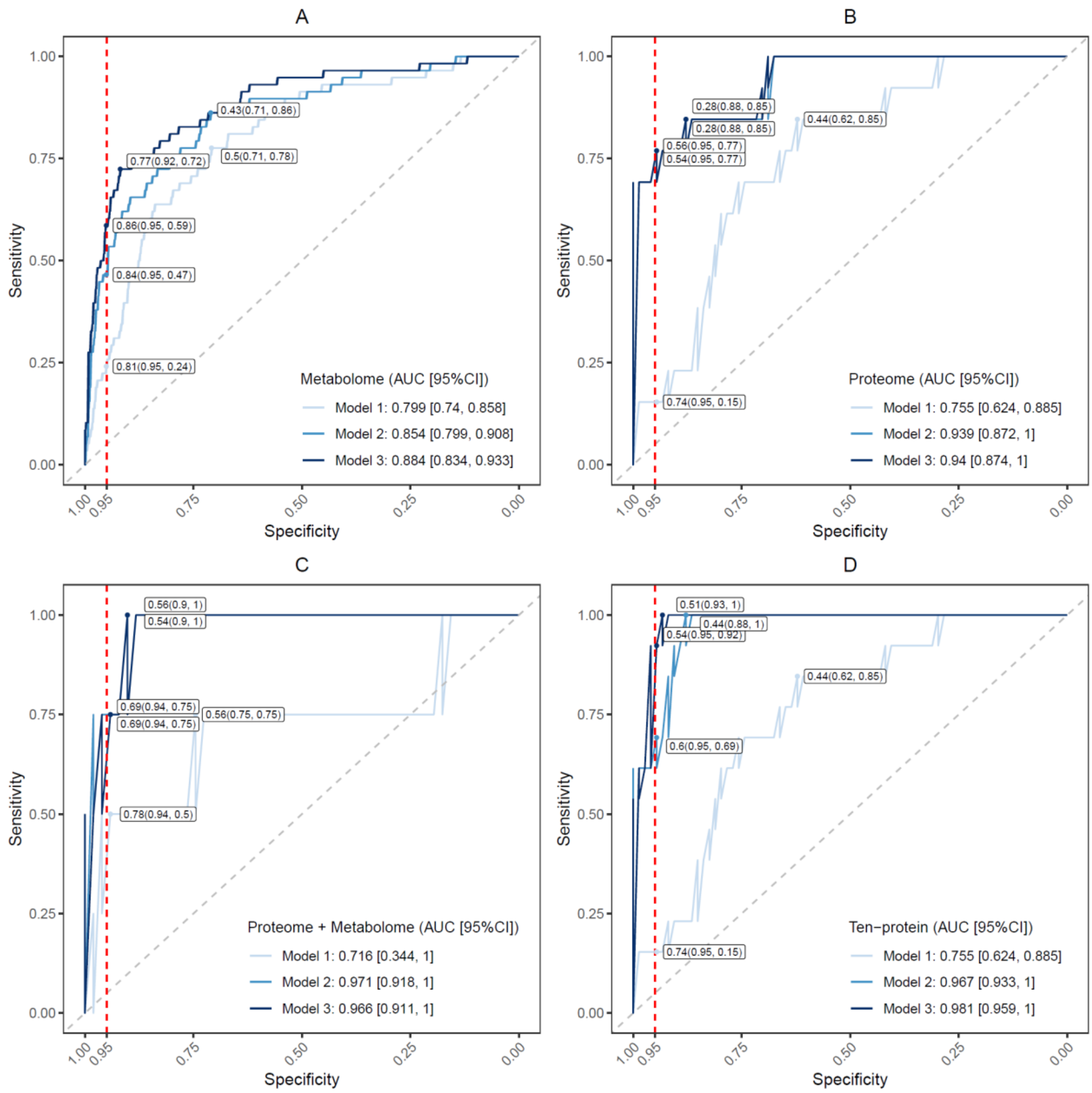

Fig. S8 Classification performance of primary models in differentiating PDFF-diagnosed ALD and MASLD. A) metabolome-related models; B) proteome-related models; C) proteome and metabolome-related models; D) ten-protein models. Variables of Model 1 include age, sex, BMI, and liver enzymes. Model 2 includes metabolites and/or proteins. Model 3 combines the variables in Model 1 and Model 2. The plots display two thresholds for each model: one for optimal specificity and sensitivity, and another for specificity set at 0.95, followed by corresponding specificity and sensitivity values. Narrow "N" pattern was presented when the number of cases was small (n≤10).

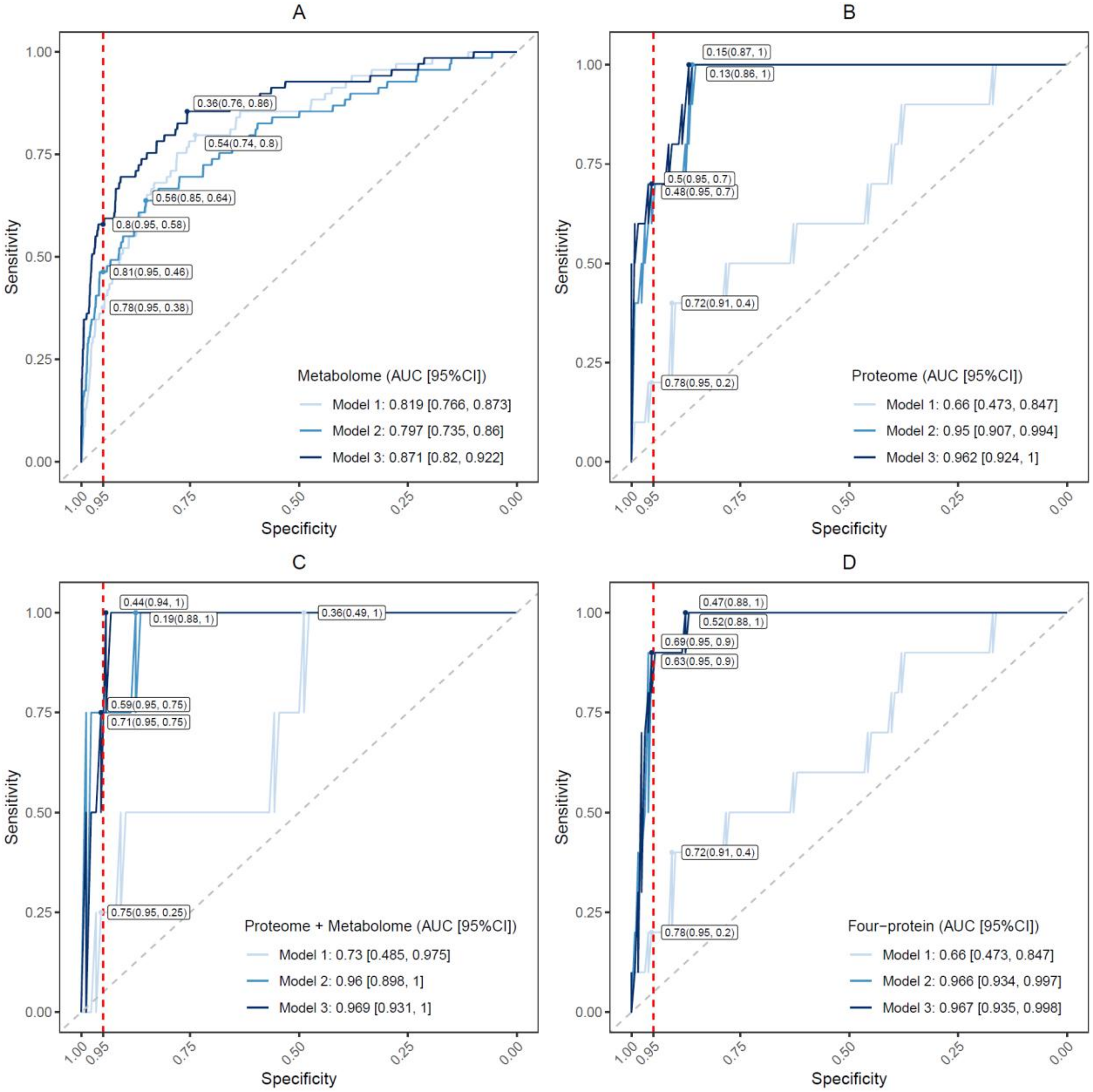

Fig. S9 Classification performance of models in differentiating ALD and MASLD based on PDFF-based diagnosis. A) metabolome-related models; B) proteome-related models; C) proteome and metabolome-related models; D) ten-protein models. Variables of Model 1 include age, sex, BMI, and liver enzymes. Model 2 includes metabolites and/or proteins. Model 3 combines the variables in Model 1 and Model 2. The plots display two thresholds for each model: one for optimal specificity and sensitivity, and another for specificity set at 0.95, followed by corresponding specificity and sensitivity values. Narrow "N" pattern was presented when the number of cases was small (n≤10).

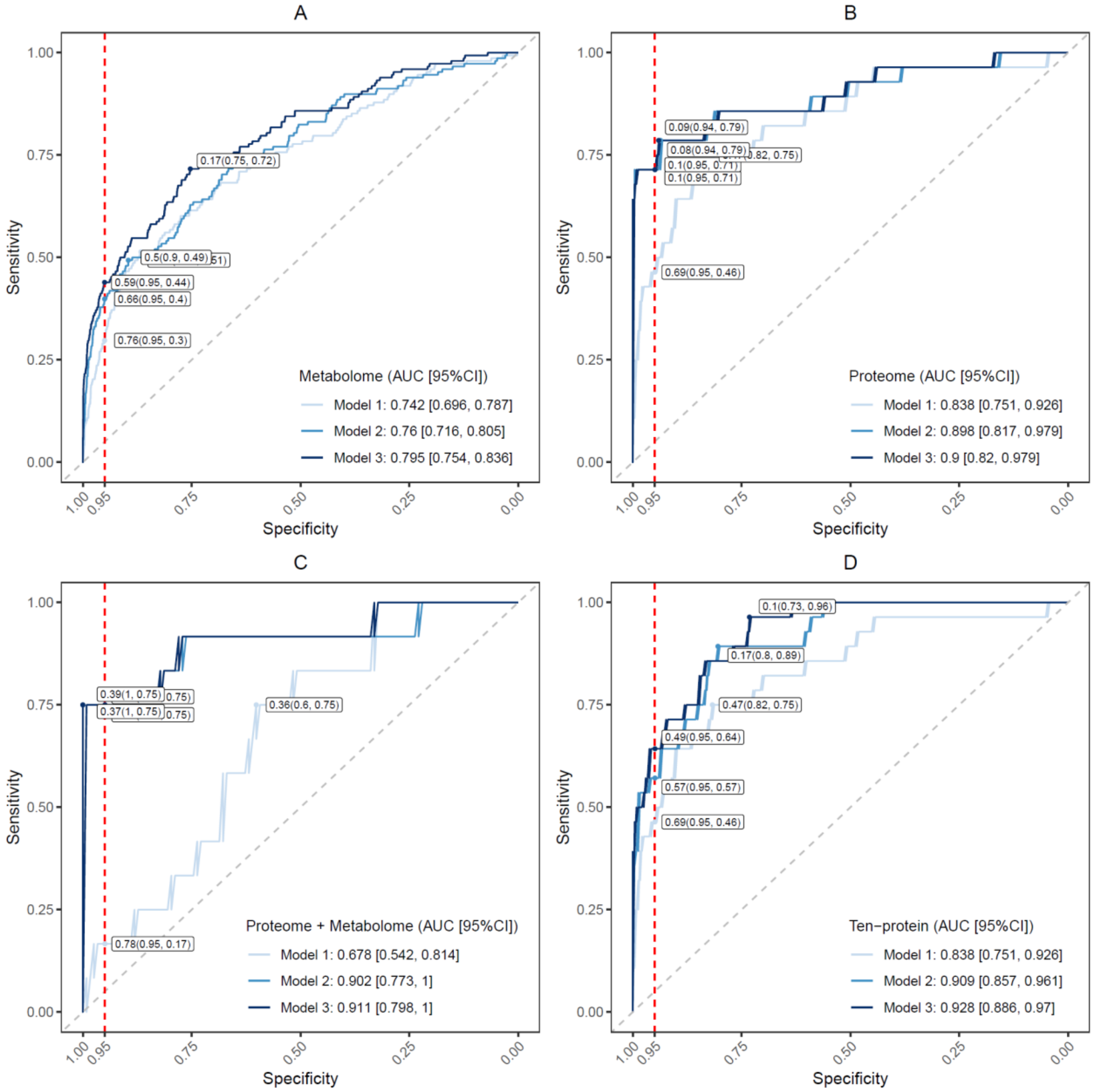

Fig. S10 Classification performance of primary models in differentiating ALD with recorded alcohol use disorders and MASLD with no alcohol intake. A) metabolome-related models; B) proteome-related models; C) proteome and metabolome-related models; D) ten-protein models. Variables of Model 1 include age, sex, BMI, and liver enzymes. Model 2 includes metabolites and/or proteins. Model 3 combines the variables in Model 1 and Model 2. The plots display two thresholds for each model: one for optimal specificity and sensitivity, and another for specificity set at 0.95, followed by corresponding specificity and sensitivity values. Narrow "N" pattern was presented when the number of cases was small (n≤10).

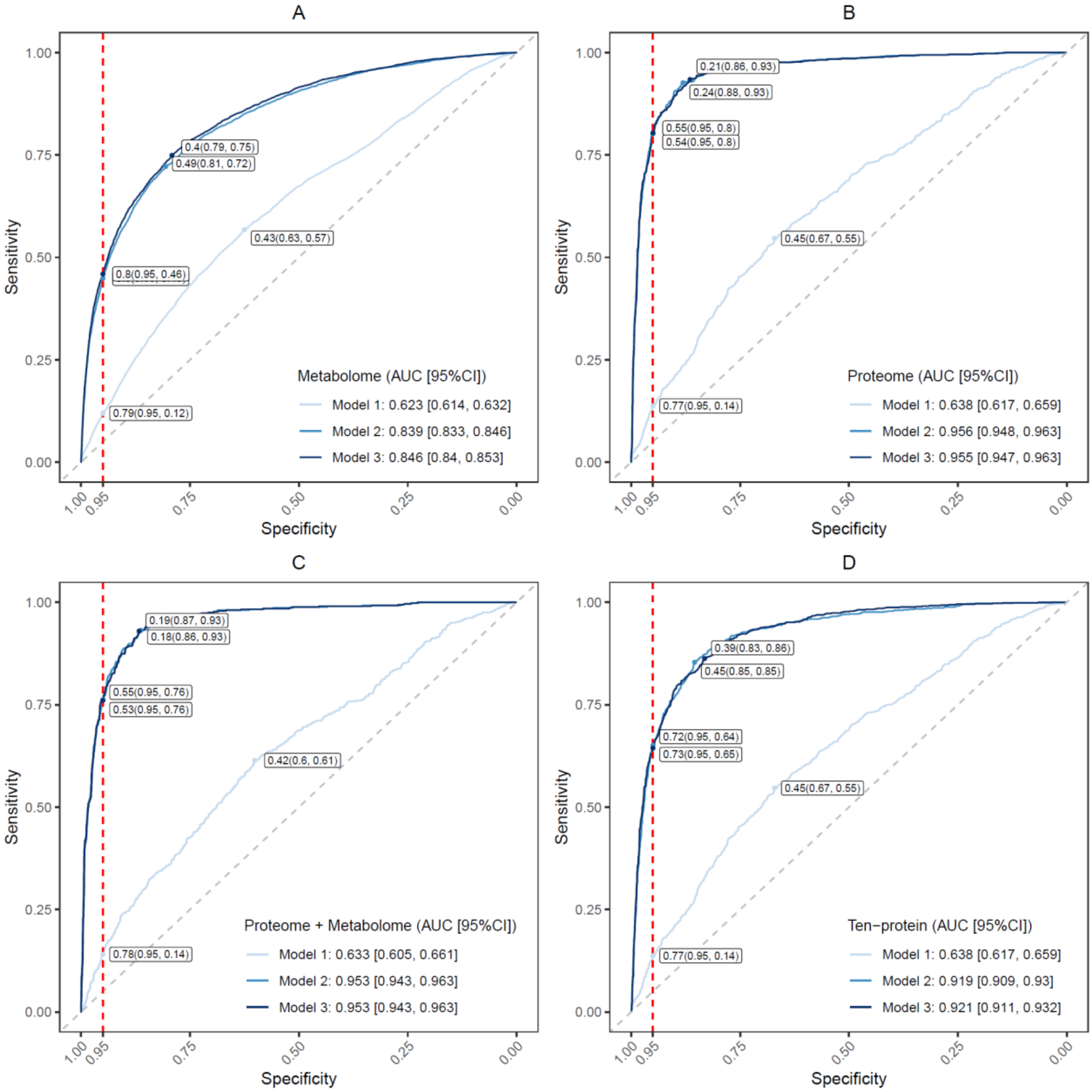

Fig. S11 Classification performance of primary models in differentiating ALD, regardless of the liver steatosis status, and MASLD. A) metabolome-related models; B) proteome-related models; C) proteome and metabolome-related models; D) ten-protein models. Variables of Model 1 include age, sex, BMI, and liver enzymes. Model 2 includes metabolites and/or proteins. Model 3 combines the variables in Model 1 and Model 2. The plots display two thresholds for each model: one for optimal specificity and sensitivity, and another for specificity set at 0.95, followed by corresponding specificity and sensitivity values.

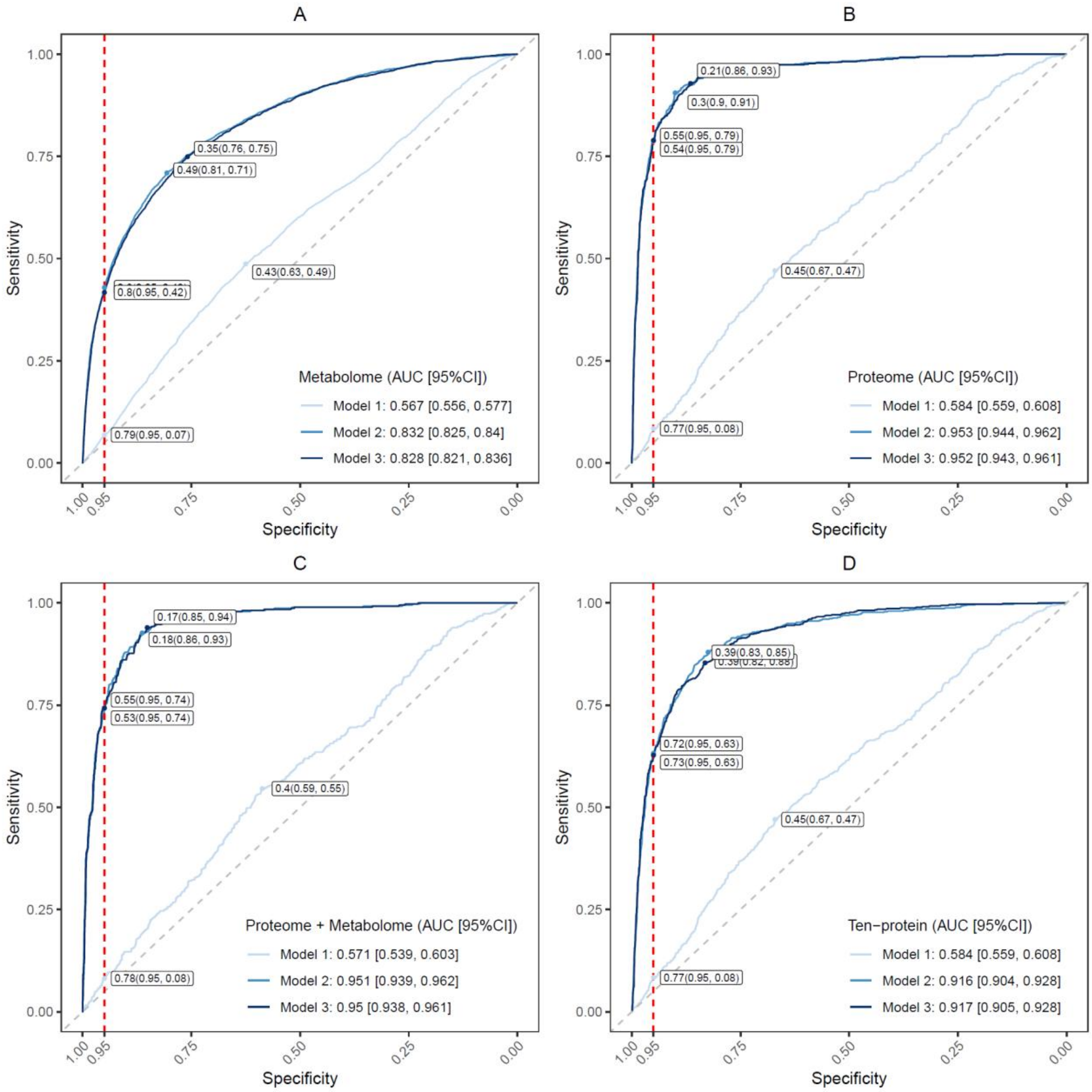

Fig. S12 Classification performance of primary models in differentiating ALD without liver steatosis and MASLD. A) metabolome-related models; B) proteome-related models; C) proteome and metabolome-related models; D) ten-protein models. Variables of Model 1 include age, sex, BMI, and liver enzymes. Model 2 includes metabolites and/or proteins. Model 3 combines the variables in Model 1 and Model 2. The plots display two thresholds for each model: one for optimal specificity and sensitivity, and another for specificity set at 0.95, followed by corresponding specificity and sensitivity values.

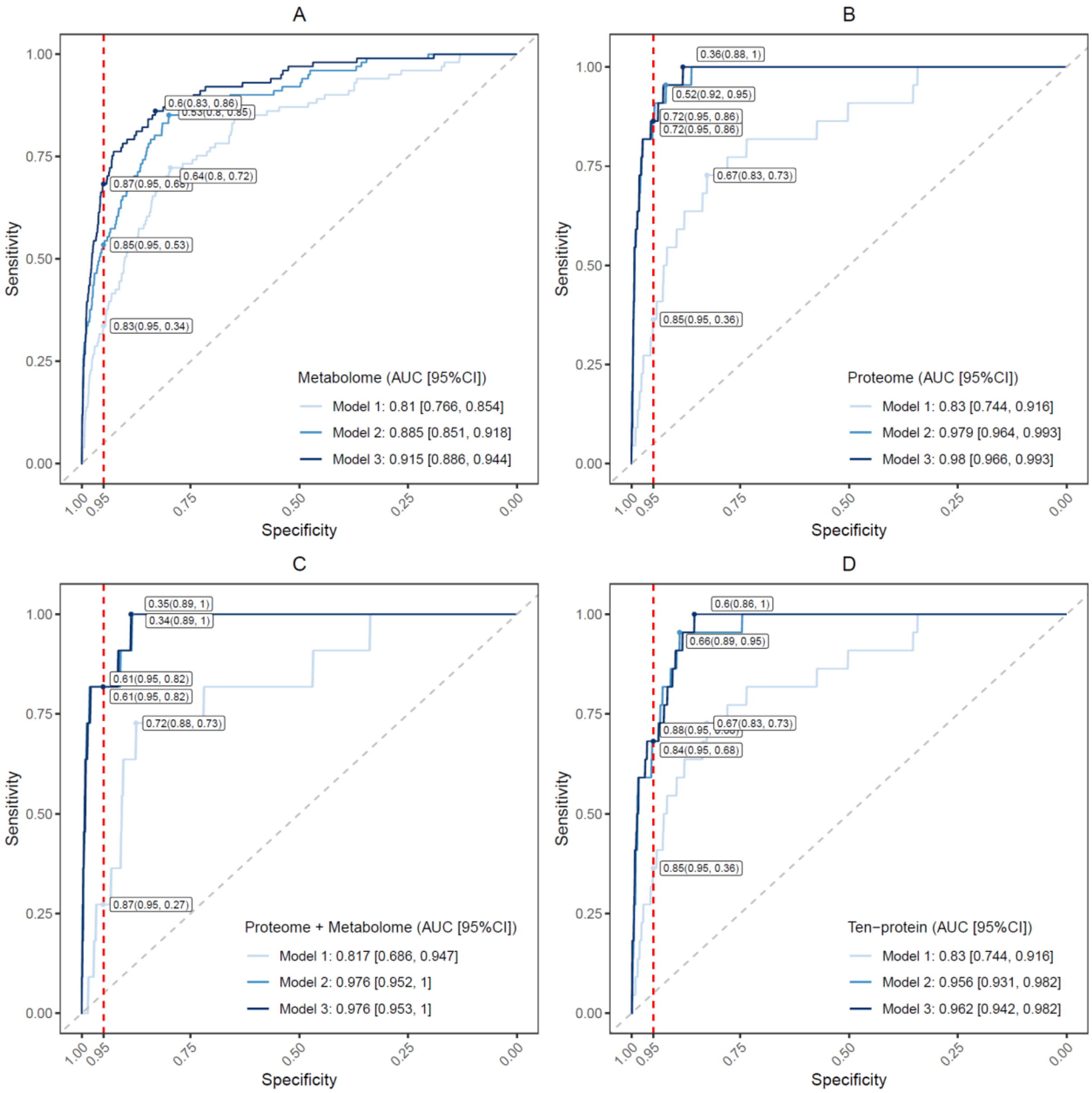

Fig. S13 Classification performance of primary models in differentiating ALD and MASLD in the non-European population in the UK Biobank. A) metabolome-related models; B) proteome-related models; C) proteome and metabolome-related models; D) ten-protein models. Variables of Model 1 include age, sex, BMI, and liver enzymes. Model 2 includes metabolites and/or proteins. Model 3 combines the variables in Model 1 and Model 2. The plots display two thresholds for each model: one for optimal specificity and sensitivity, and another for specificity set at 0.95, followed by corresponding specificity and sensitivity values.

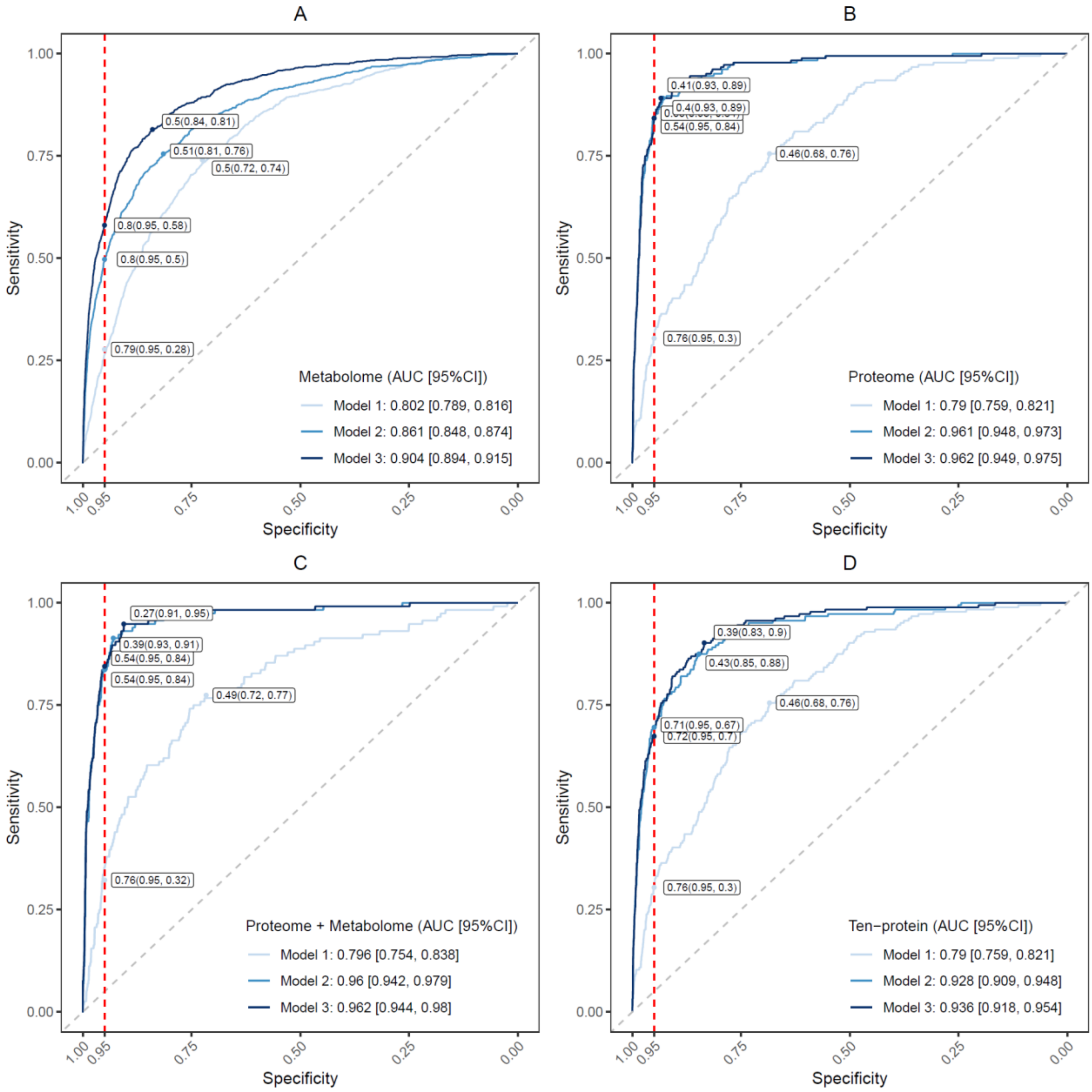

Fig. S14 Classification performance of primary models in differentiating ALD and MASLD in participants without any missing values. A) metabolome-related models; B) proteome-related models; C) proteome and metabolome-related models; D) ten-protein models. Variables of Model 1 include age, sex, BMI, and liver enzymes. Model 2 includes metabolites and/or proteins. Model 3 combines the variables in Model 1 and Model 2. The plots display two thresholds for each model: one for optimal specificity and sensitivity, and another for specificity set at 0.95, followed by corresponding specificity and sensitivity values.

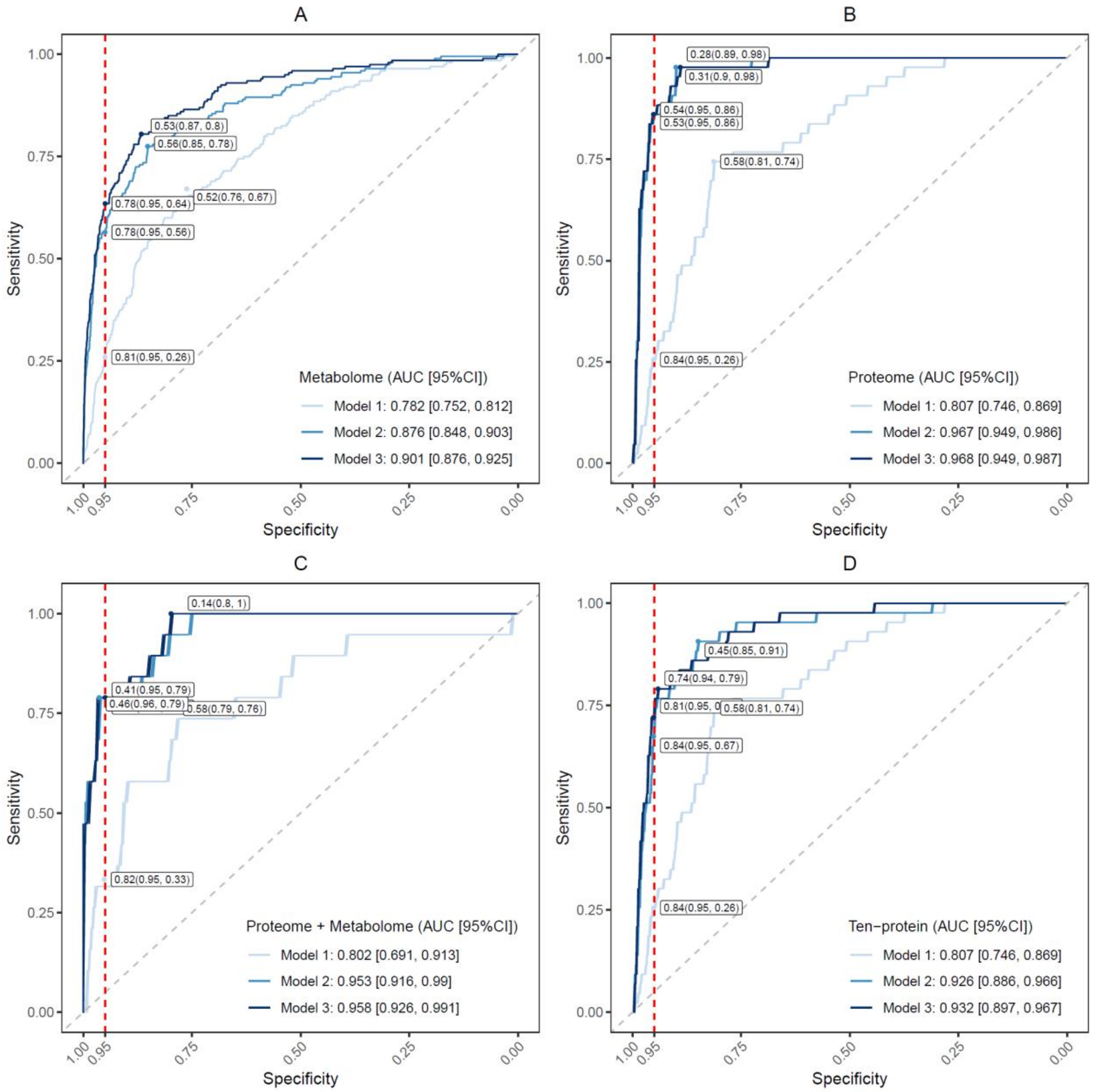

Fig. S15 Classification performance of primary models in differentiating ALD and MASLD in participants with at least one imputed value. A) metabolome-related models; B) proteome-related models; C) proteome and metabolome-related models; D) ten-protein models. Variables of Model 1 include age, sex, BMI, and liver enzymes. Model 2 includes metabolites and/or proteins. Model 3 combines the variables in Model 1 and Model 2. The plots display two thresholds for each model: one for optimal specificity and sensitivity, and another for specificity set at 0.95, followed by corresponding specificity and sensitivity values.

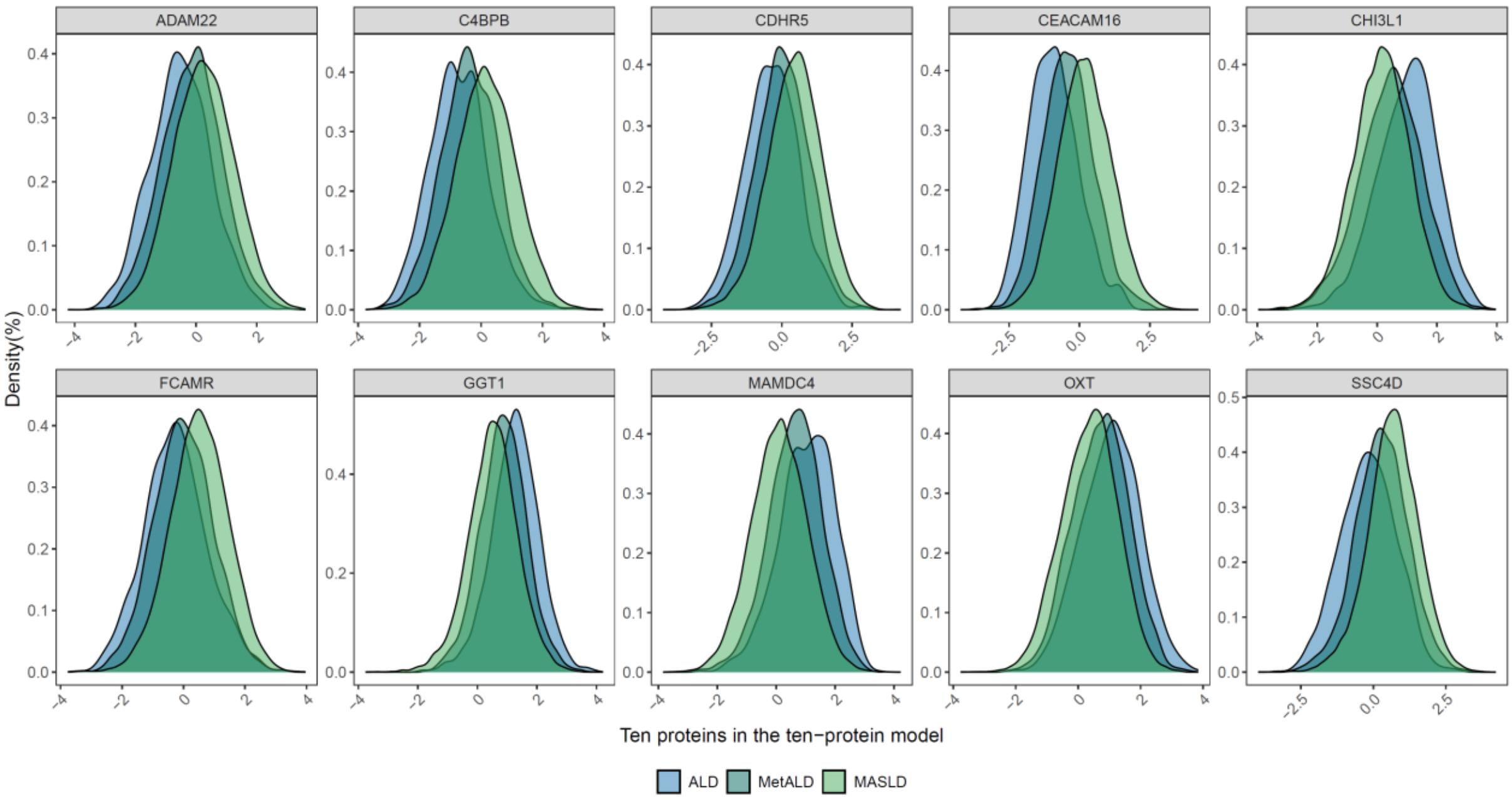

Fig. S16 Distribution of the ten proteins included in the ten-protein model across SLD types. X-axis shows the transformed values of the proteins.

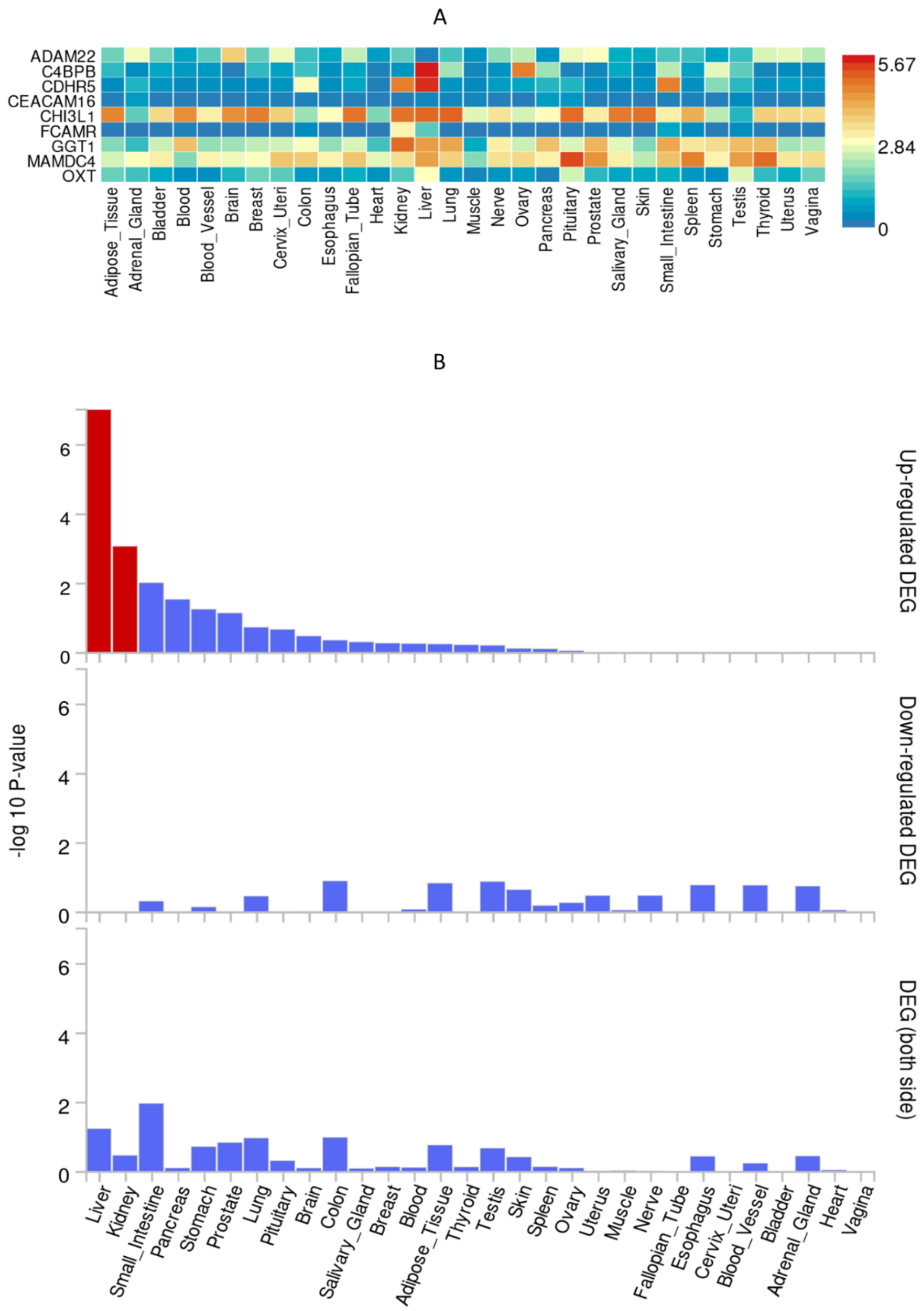

Fig. S17 Gene expression levels in different tissues (A) and tissue specificity (B) of the encoding genes of the ten identified proteins. The figures were generated based on the default pipeline using FUMA with Gex (v8) dataset in 30 tissue types (K Watanabe, et al. Nat Commun, 2019). The colours in Figure A show the average expression per label (log2 transformed). In Figure B, significantly enriched differentially expressed genes (DEG) sets ($p_{bon} < 0.05$) are highlighted in red.

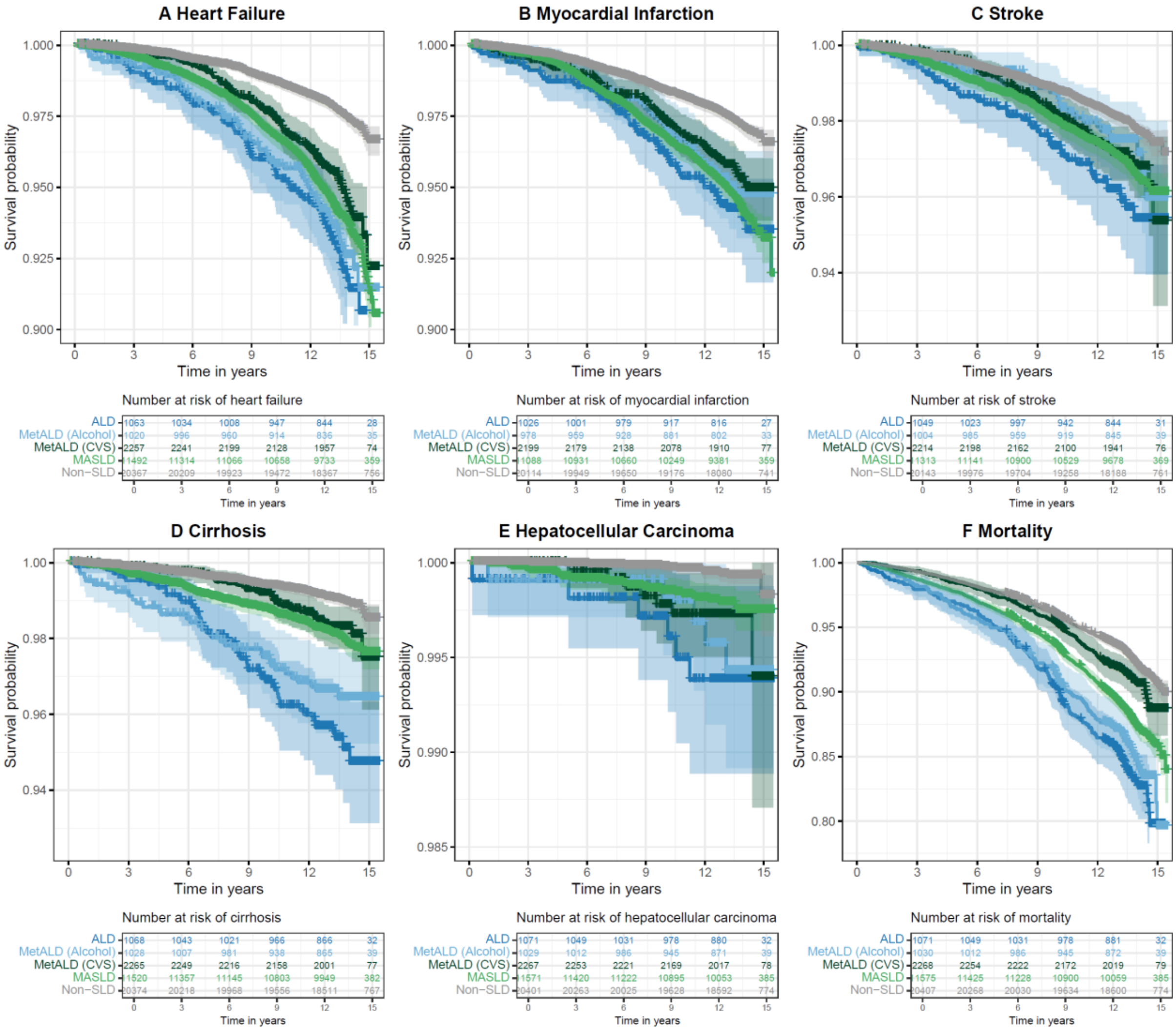

Fig. S18 Survival curves of major outcomes in SLD subtypes, MetALD subtypes and non-SLD controls. MetALD (Alcohol): alcohol-predominant MetALD group; MetALD (CVS): cardiometabolic-predominant MetALD group.

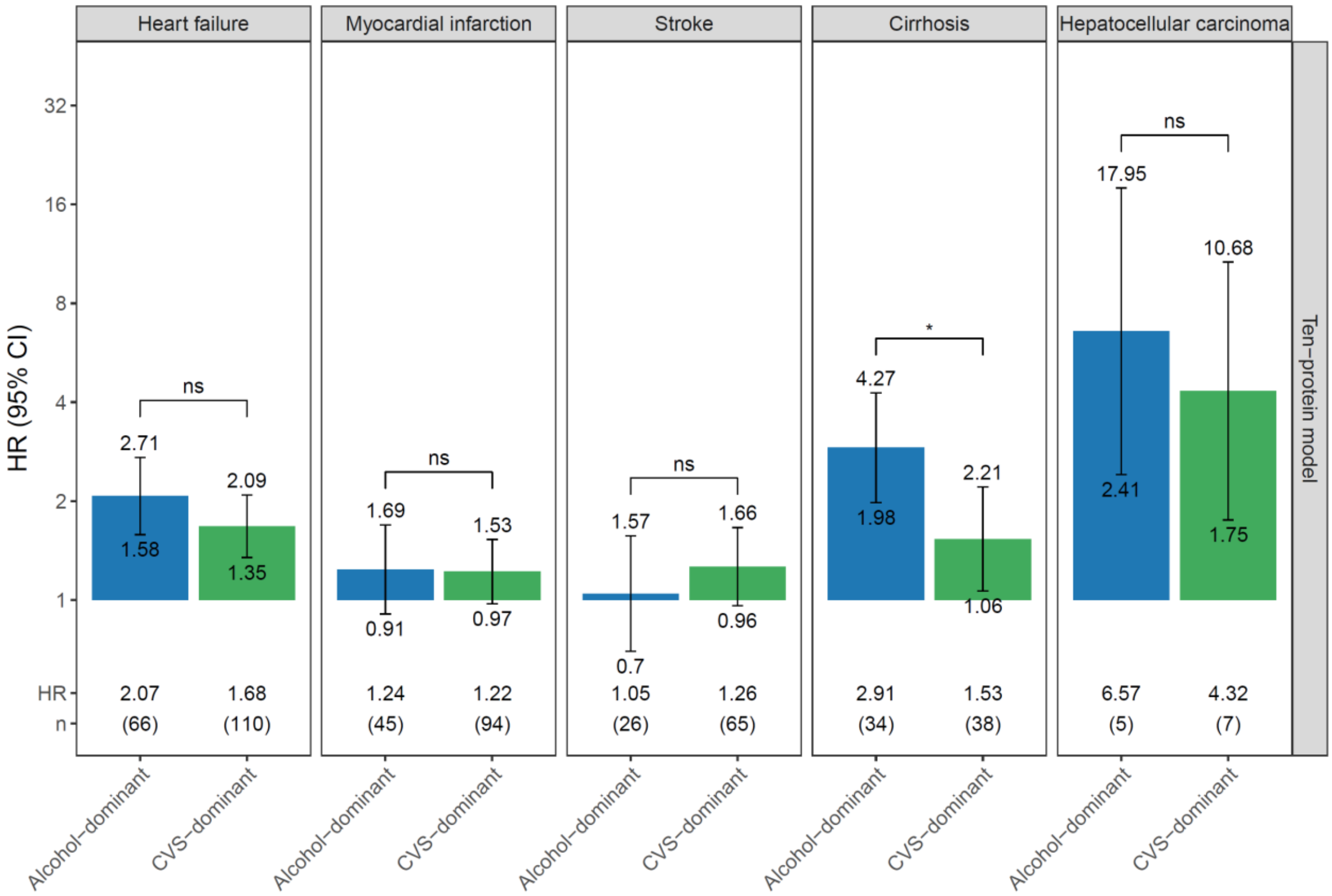

Fig. S19 HR of major outcomes in MetALD subgroup patients compared to non-SLD controls, accounting for competing risk of mortality. Cox regression was performed with adjustments for age, sex, and fasting time. Adjustments were made for age, sex, and fasting time. The HR values are positioned beneath the bar plots, accompanied by 95% CI adjacent to the error bars. Number of incident outcome events in each case group is denoted by "n" in the brackets. CVS: cardiometabolic. *** p<0.0001, ** p<0.001, * $p<8.3\times10^{-3}$, · p<0.05, ns $p\geq0.05$.

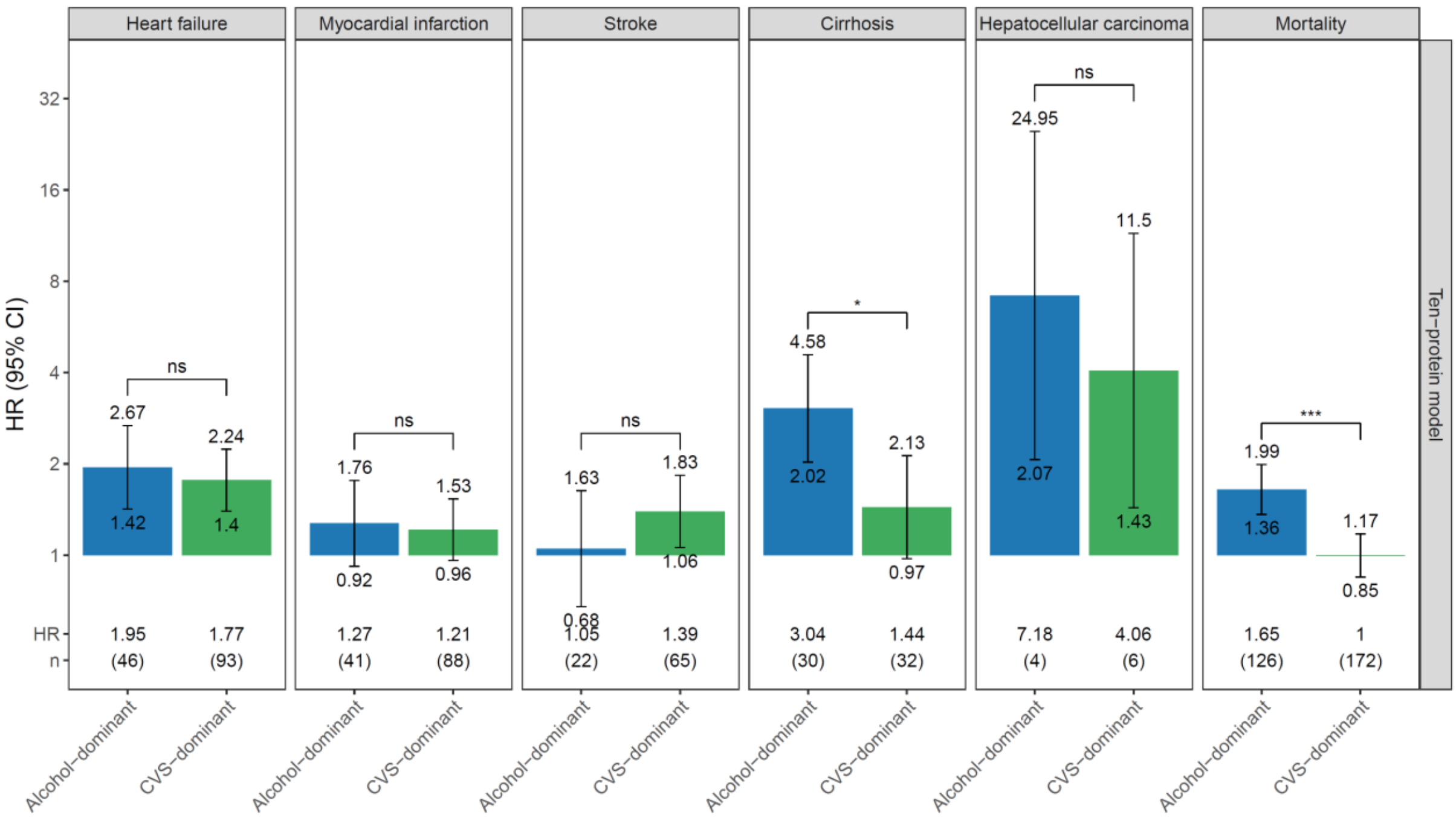

Fig. S20 Sensitivity analysis: HR of major outcomes in MetALD subgroup patients compared to non-SLD controls. Patients with any major outcomes at baseline were excluded from the survival analysis. Cox regression was performed with adjustments for age, sex, and fasting time. The HR values are positioned beneath the bar plots, accompanied by 95% CI adjacent to the error bars. Number of incident outcome events in each case group is denoted by "n" in the brackets. CVS: cardiometabolic. *** p<0.0001, ** p<0.001, * $p<8.3\times10^{-3}$, · p<0.05, ns $p\geq0.05$.

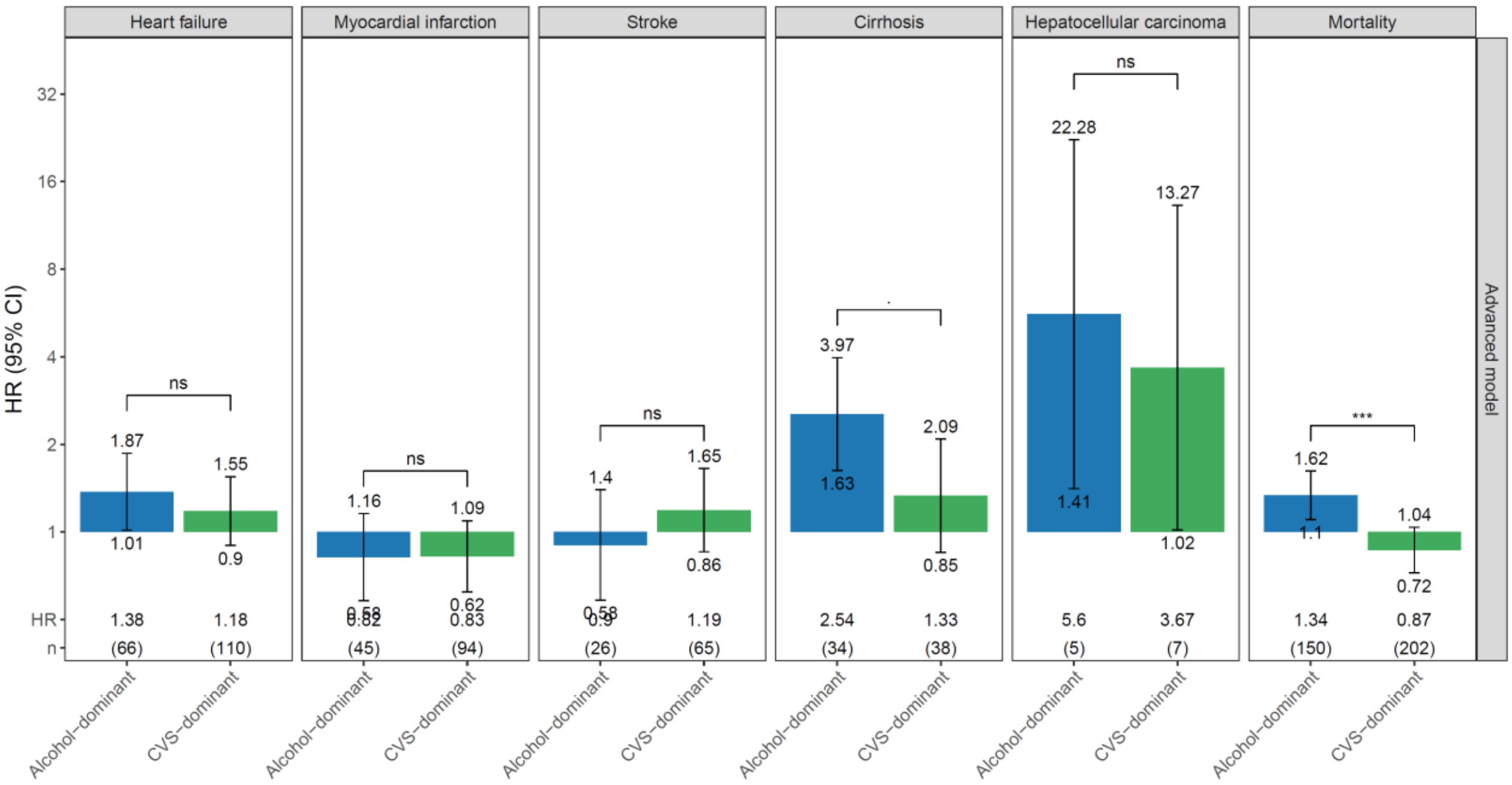

Fig. S21 HR of major outcomes in MetALD subgroup patients compared to non-SLD controls in advanced model. Cox regression was performed with advanced adjustments for age, sex, fasting time, BMI, lifestyle factors (smoking status, pack years, education, and physical activity), baseline cardiometabolic comorbidities (T2D, and hypertension) and medications (anti-diabetes, anti-hypertensives and lipid lowering medications). The HR values are positioned beneath the bar plots, accompanied by 95% CI adjacent to the error bars. Number of incident outcome events in each case group is denoted by "n" in the brackets. CVS: cardiometabolic. *** $p<0.0001$, ** $p<0.001$, * $p<8.3\times10^{-3}$, · $p<0.05$, ns $p\geq0.05$.

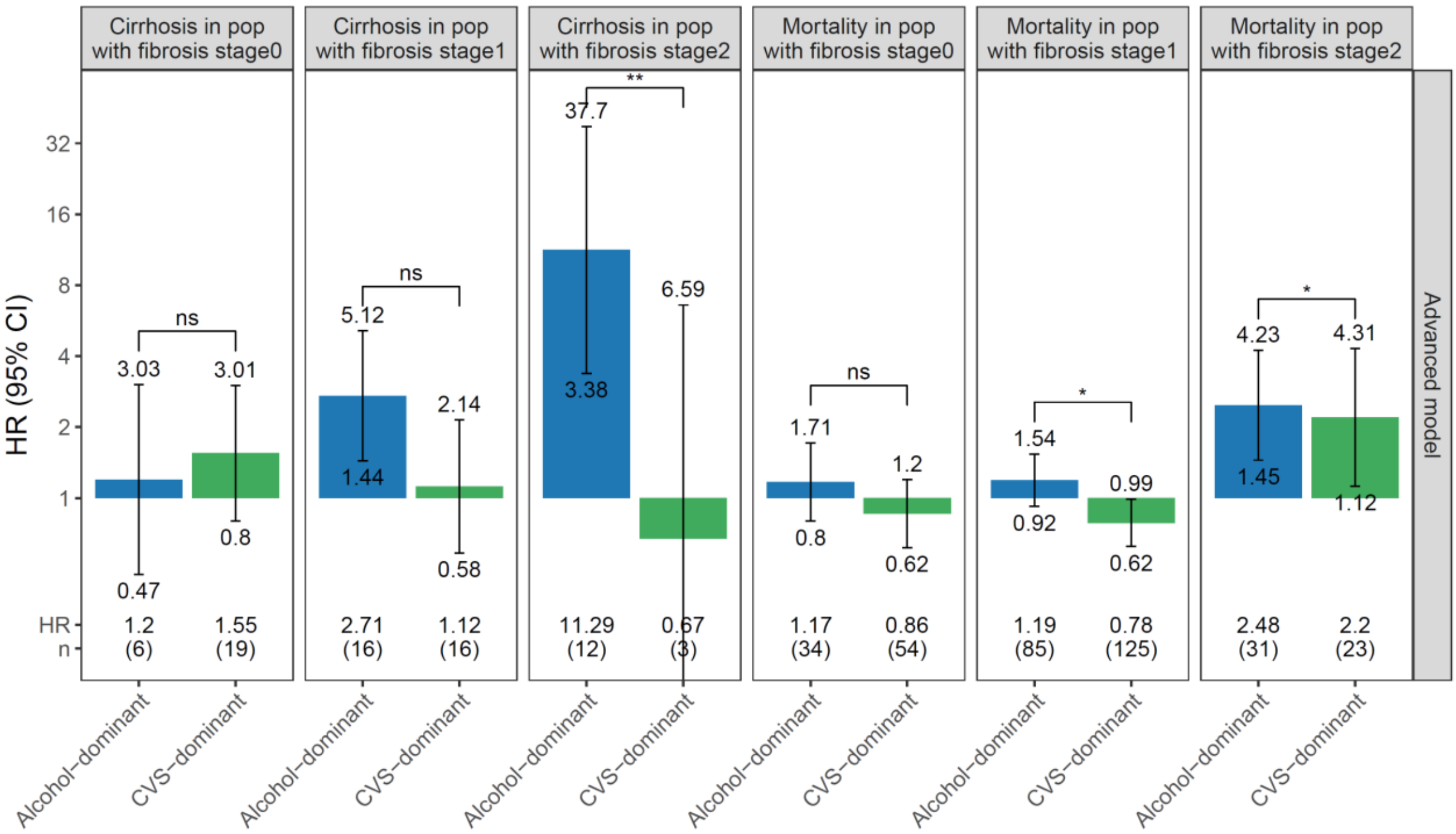

Fig. S22 HR of cirrhosis and mortality in MetALD subgroups, compared to non-SLD controls, stratified by fibrosis stages, in advanced model. Cox regression was performed with advanced adjustments for age, sex, fasting time, BMI, lifestyle factors (smoking status, pack years, education, and physical activity), baseline cardiometabolic comorbidities (T2D, and hypertension) and medications (anti-diabetes, anti-hypertensives and lipid lowering medications). The HR values are positioned beneath the bar plots, accompanied by 95% CI adjacent to the error bars. Number of incident outcome events in each case group is denoted by "n" in the brackets. CVS: cardiometabolic. *** p<0.0001, ** p<0.001, * p<0.025, · p<0.05, ns p≥0.05.

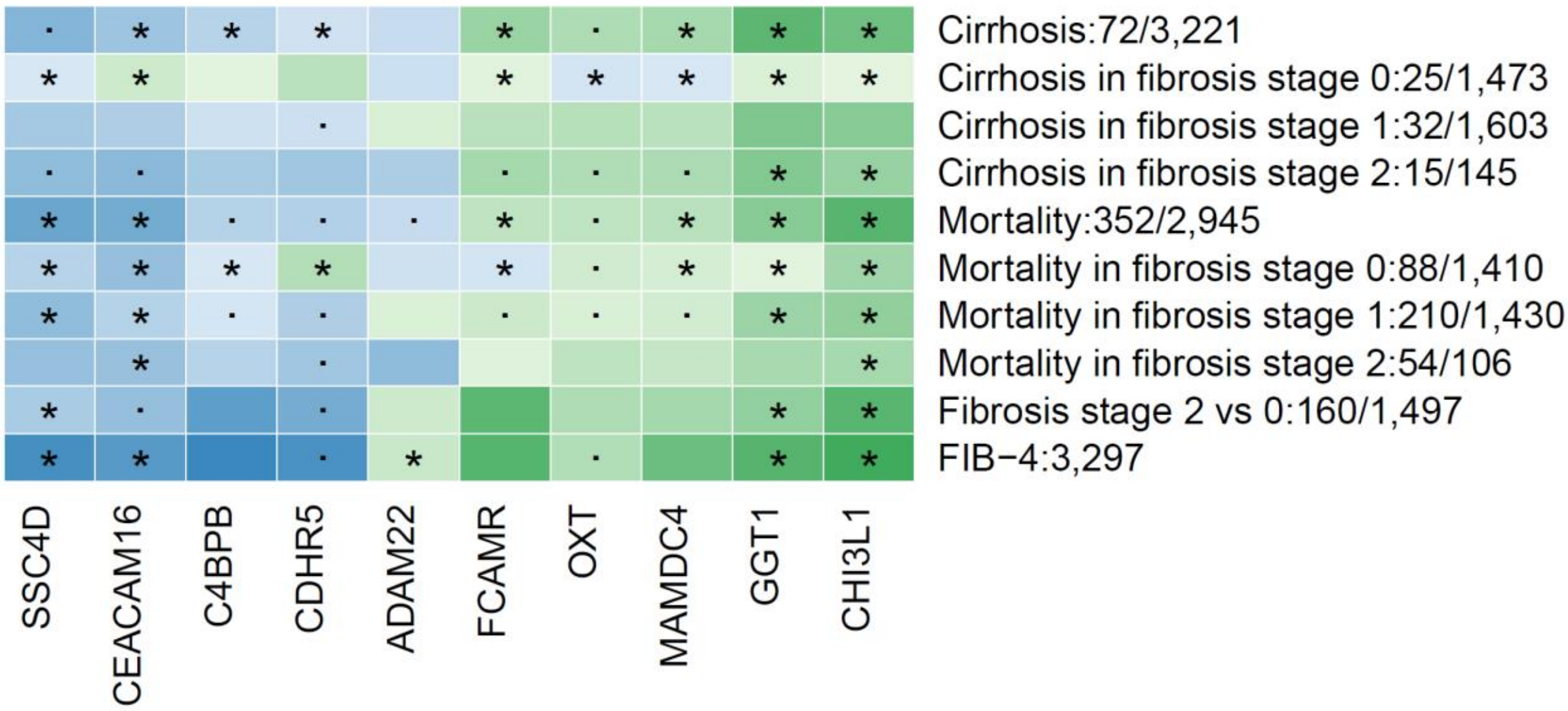

Fig. S23 Association of the ten identified proteins with cirrhosis, mortality and fibrosis traits and stratification in MetALD patients. Sample sizes for the analyses (cases/controls or total samples) are listed. Logistic/linear regression was performed with adjustment for age, sex, and fasting time. Green indicates positive, blue indicates negative associations, with colour depth reflecting the magnitude of the z-score. Hierarchical clustering was used to group the proteins. * $p<2.5\times10^{-3}$, · $p<0.05$.

Supplementary Tables (Excel file provided)

Supplementary Table 1 Disease definition in the UK Biobank

Supplementary Table 2 Definition of MASLD related traits and covariates used in the study

Supplementary Table 3 Map of studied metabolites

Supplementary Table 4 Map of studied proteins
The protein label presents the annotated proteins and measurement panels. *Ensembl shows the Ensembl names of the first protein.

Supplementary Table 5 Significant association of metabolites and ALD versus MASLD (reference group) using FLI-based diagnosis
Losgitic regression was performed with adjustment for age, sex, and fasting time. *When P less than 1.0E-315, 0 is shown in the table.

Supplementary Table 6 Significant association of proteins and ALD versus MASLD (reference group) using FLI-based diagnosis
Logistic regression was performed with adjustment for age, sex, and fasting time.

Supplementary Table 7 Significant association of metabolites and ALD versus MASLD (reference group) using PDFF-based diagnosis
Logistic regression was performed with adjustment for age, sex, and fasting time.

Supplementary Table 8 Significant association of proteins and ALD versus MASLD (reference group) using PDFF-based diagnosis
Logistic regression was performed with adjustment for age, sex, and fasting time.

Supplementary Table 9 The association of proteins and alcohol grams per week (n=41,767)
Linear regression was performed with adjustment for age, sex, and fasting time. * When P less than 1.0E-315, 0 is shown in the table. Adjusted for age, sex, and fasting time.

Supplementary Table 10 Association of MetALD subgroups and liver fibrosis variables
Alcohol-predominant group was coded as 1; cardiometabolic-predominant group was coded as 0. Logistic regression was performed. Basic model adjusted for age, sex and fasting time. Advanced model additionally adjusted for BMI, lifestyle factors (smoking status, pack years, education, and physical activity), baseline cardiometabolic comorbidities (type 2 diabetes, and hypertension) and medications (anti-diabetes, anti-hypertensives and lipid lowering medications)

References

1. Wright, M. N. & Ziegler, A. ranger: A fast implementation of random forests for high dimensional data in C++ and R. *Journal of statistical software* **77**, 1–17 (2017).
2. Stekhoven, D. J. & Bühlmann, P. MissForest--non-parametric missing value imputation for mixed-type data. *Bioinformatics* **28**, 112–118 (2012).
3. Van Buuren, S. & Groothuis-Oudshoorn, K. mice: Multivariate imputation by chained equations in R. *Journal of statistical software* **45**, 1–67 (2011).
4. European Association for the Study of the Liver (EASL), European Association for the Study of Diabetes (EASD), & European Association for the Study of Obesity (EASO). EASL-EASD-EASO Clinical Practice Guidelines on the management of metabolic dysfunction-associated steatotic liver disease (MASLD). *J Hepatol* **81**, 492–542 (2024).
