# Supplementary material for: Dissecting metabolic dysfunction- and alcohol-associated liver disease (MetALD) using proteomic and metabolomic profiles
Source: J Hepatol. Author manuscript; Available in PMC 2026 Sep 4. (PMC7619437; doi:10.1016/j.jhep.2025.05.026)
Supplement: Supplementary file 3 [file EMS217801-supplement-Multimedia_component_2.docx]

**Journal of Hepatology**

**CTAT methods**

Tables for a “Complete, Transparent, Accurate and Timely account” (CTAT) are now mandatory for all revised submissions. The aim is to enhance the reproducibility of methods.

- Only include the parts relevant to your study
- Refer to the CTAT in the main text as ‘Supplementary CTAT Table’
- Do not add subheadings
- Add as many rows as needed to include all information
- Only include one item per row

**If the CTAT form is not relevant to your study, please outline the reasons why:**

Only 1.6, 1.7 and 1.9 are relevant to the current study, and the answers have been provided below.

1.1 **Antibodies**

| **Name** | **Citation** | **Supplier** | **Cat no.** | **Clone no.** |
| --- | --- | --- | --- | --- |
| **-** |  |  |  |  |

1.2 **Cell lines**

| **Name** | **Citation** | **Supplier** | **Cat no.** | **Passage no.** | **Authentication test method** |
| --- | --- | --- | --- | --- | --- |
| **-** |  |  |  |  |  |

1.3 **Organisms**

| **Name** | **Citation** | **Supplier** | **Strain** | **Sex** | **Age** | **Overall n number** |
| --- | --- | --- | --- | --- | --- | --- |
| **-** |  |  |  |  |  |  |

1.4 **Sequence based reagents**

| **Name** | **Sequence** | **Supplier** |
| --- | --- | --- |
| **-** |  |  |

1.5 **Biological samples**

| **Description** | **Source** | **Identifier** |
| --- | --- | --- |
| **-** |  |  |

1.6 **Deposited data**

| **Name of repository** | **Identifier** | **Link** |
| --- | --- | --- |
| **UK Biobank** | **53639 and 65851** | **http://www.ukbiobank.ac.uk/** |

1.7 **Software**

| **Software name** | **Manufacturer** | **Version** |
| --- | --- | --- |
| **R statistical software** | **R project** | **4.0.3** |

1.8 **Other (e.g. drugs, proteins, vectors etc.)**

| **-** |  |  |
| --- | --- | --- |
|  |  |  |

1.9 **Please provide the details of the corresponding methods author for the manuscript:**

**Dr. Jun Liu, Nuffield Department of Population Health, University of Oxford, email:** [**jun.liu@ndph.ox.ac.uk**](mailto:jun.liu@ndph.ox.ac.uk)

**2.0	Please confirm for randomised controlled trials all versions of the clinical protocol are included in the submission. These will be published online as supplementary information.**

**Not applicable**
